# Smoking status and cessation duration in relation to the progression of cardio-renal-metabolic multimorbidity: a prospective cohort study from the UK Biobank

**DOI:** 10.1186/s13690-026-01846-x

**Published:** 2026-02-03

**Authors:** Xinhui Liu, Shuo Wu, Heng Zhang, Fuzhong Xue

**Affiliations:** 1 Department of Emergency Medicine, Qilu Hospital of Shandong University, Jinan, China; 2 Shandong Provincial Clinical Research Center for Emergency and Critical Care Medicine, Institute of Emergency and Critical Care Medicine of Shandong University, Chest Pain Center, Qilu Hospital of Shandong University, Jinan, China; 3 Department of Neurology, Shandong Provincial Hospital Affiliated to Shandong First Medical University, Jinan, China; 4 Department of Biostatistics, School of Public Health, Cheeloo College of Medicine, Shandong University, Jinan, China; 5 Healthcare Big Data Research Institute, School of Public Health, Cheeloo College of Medicine, Shandong University, Jinan, China; 6 Qilu Hospital, Cheeloo College of Medicine, Shandong University, Jinan, China

**Keywords:** Smoking cessation, Cardio-renal-metabolic multimorbidity, Multi State model

## Abstract

**Background:**

This study aimed to investigate the association of smoking status and years since cessation with the onset, progression, and prognosis of cardio-renal-metabolic (CRM) multimorbidity (CRMM).

**Methods:**

This study included participants from the UK Biobank who were free of CRM disease at baseline. Covariates adjusted Cox proportional hazards models were employed to evaluate the associations of smoking status and years since smoking cessation with the risks of individual CRM diseases, including ischemic heart disease (IHD), stroke, type 2 diabetes (T2D), and chronic kidney disease (CKD), as well as with each state in CRMM progression, including first CRM disease (FCRMD), CRMM (defined as the occurrence of two or more CRM diseases), and death. Multi-state models were used to analyze the associations between smoking-related behaviors and CRMM progression. The effects of smoking cessation were further explored within subgroups according to sex, age at smoking initiation, smoking duration, smoking intensity, and genetic risk scores for individual CRM diseases.

**Results:**

In total, 356,071 participants (median age 57 years; 44.9% male) who were free of CRM disease (healthy) at baseline and had complete information on smoking status were included. During a median follow-up of 13.6 years, 56,786 participants developed a FCRMD, and 11,508 progressed to CRMM, of whom 2,796 subsequently died. Across all transitions from healthy to FCRMD, then to CRMM, and ultimately to death, current smoking had a greater impact on transitions leading to mortality. Compared with never smokers, current smokers had an adjusted hazard ratio of 1.44 (95% *CI*: 1.40–1.47) for the transition from healthy to FCRMD and 2.49 (95% *CI*: 2.38–2.60) for the transition from healthy to death. Approximately 25 years of smoking cessation were required for risks across all transitions in CRMM progression among former smokers to became not significantly different from those of never smokers. Compared with current smokers, former smokers experienced significantly lower risks for transitions leading to death shortly after cessation, whereas risk reductions for the transitions from healthy to FCRMD and from FCRMD to CRMM were not observed until more than 5 and 20 years after cessation, respectively. When disease-specific transitions were further considered, longer post-cessation periods were required to achieve significant risk reductions for transitions from healthy to T2D or CKD and from IHD or T2D to death, compared with current smokers. The effects of smoking cessation on CRMM progression varied by sex and previous smoking behavior, but not by genetic susceptibility to specific CRM diseases.

**Conclusion:**

Smoking has substantial but varied impacts across transitions in CRMM progression and disease-specific pathways. Long-term smoking cessation is an important strategy for reducing the risk of CRMM onset and progression. Individuals with specific prior smoking patterns (e.g., male or heavy smokers) and those at certain transition states warrant particular attention during the short-term period following smoking cessation.

**Supplementary Information:**

The online version contains supplementary material available at 10.1186/s13690-026-01846-x.

**Table:** 

**Text box 1**. Contributions to the Literature
• Cardio-renal-metabolic multimorbidity (CRMM) is an increasingly important public health challenge, yet evidence on how smoking behavior and cessation duration influence its progression remains limited.
• Early and sustained smoking cessation should be prioritized across the full continuum of CRMM to reduce disease progression and premature mortality.
• The early post-cessation period represents a critical window during which former smokers at specific disease stages require enhanced clinical monitoring and risk management.
• High-risk subgroups, such as women and individuals with early smoking initiation, may derive substantial short-term benefits from cessation and should be prioritized in targeted cessation programs.

## Background

Cardio-renal-metabolic (CRM) conditions are an important public health concern and have received increasing attention in clinical guidance documents [1, 2]. Owing to their close physiological interconnections, CRM diseases, including cardiovascular disease (CVD), type 2 diabetes (T2D), and chronic kidney disease (CKD), often coexist [3–5]. The presence of one CRM disease tends to accelerate the development of others, collectively increasing adverse outcomes. The coexistence of two or more CRM diseases is referred to as CRM multimorbidity (CRMM) [6, 7]. Compared with a single CRM disease, the progression of CRMM is associated with a markedly higher mortality worldwide [8]. It is imperative to implement interventions to delay the progression of CRMM.

Smoking is an important modifiable lifestyle factor that substantially contributes to the risk of individual CRM disease and mortality [9–11]. According to the World Health Organization, tobacco use is responsible for over 7 million deaths each year and for considerable disability from tobacco-related diseases [12]. Among lifestyle factors, smoking was also identified as one of the leading contributors (19.88% to 38.10%) to overall CRMM progression [6]. Nevertheless, existing evidence indicates that the risk of progression of individual cardiometabolic diseases and mortality associated with smoking can be largely reversed through smoking cessation [13–15]. Tobacco control policies have been implemented at both global and regional levels, and clinical guidelines increasingly highlight smoking prevention and cessation as essential strategies for both primary and secondary prevention of individual cardiometabolic diseases [16]. According to the U.S. Centers for Disease Control and Prevention, an increasing number of people have successfully quit smoking in recent years [17]. Clarifying the associations of smoking behaviors, particularly smoking cessation, with CRMM progression not only supports the advancement of tobacco control policies, but also provides important guidance for precise risk monitoring among former smokers.

Most studies evaluated the risk of cardiometabolic disease or multimorbidity among former smokers using smoking status as a single indicator, without accounting for the complexity of cessation behavior and how risk varies with time since cessation and smoking history [5, 6, 18]. However, the temporal trend of risk reduction after smoking cessation differs substantially across diseases, when compared with current smokers or never smokers [19, 20]. Additionally, for some diseases, existing evidence suggests that changes in risk after smoking cessation are influenced by previous smoking status, such as cumulative smoking amount [21]. The disease risks faced by former smokers have not yet been fully addressed in current clinical guidelines or practice. Even the recent cardiovascular risk stratification tools (such as the PREVENT equations released in 2024 for long-term cardiovascular risk prediction [22]) do not distinguish between never smokers and former smokers, nor do the Pooled Cohort Equations [16], the 10-year risk of ASCVD calculator recommended in the American Heart Association (AHA) framework for staging cardiovascular–kidney–metabolic syndrome [1]. This may lead to substantial underestimation of the disease-specific risks of former smokers. Existing studies on the health risks of smoking cessation mainly focus on individual CRM diseases [19, 21, 23, 24], without systematically evaluating the associations between smoking cessation and the onset, progression, and prognosis of CRMM. The risk of transitions in CRMM progression may also vary among former smokers depending on the duration since smoking cessation and the extent of prior smoking exposure, including cumulative lifetime exposure, which warrants further investigation.

In this study, based on data from over 350,000 UK Biobank participants with a median follow-up of approximately 14 years, we explored the associations of smoking status and years since smoking cessation with the longitudinal progression of CRMM, including transitions from being free of CRM disease to first CRM disease (FCRMD), subsequently to CRMM, and finally to death. We further investigated the effects of smoking-related traits across all disease-specific transitions when considering individual FCRMDs. We also conducted subgroup analyses across sex, previous smoking behaviors, and genetic risk for each individual CRM disease to identify population that show significantly heterogeneity in the effects of smoking cessation.

## Methods

### Study population

UK Biobank is a large-scale prospective study which recruited over 500,000 participants aged 40–69 years across the UK between 2006–2010, with longitudinal follow-up for health-related outcomes. Detailed study design and methods have been described elsewhere [25]. Ethical approval was obtained from the North West Multicenter Research Ethical Committee, and all participants provided written informed consent.

**Fig. 1:**
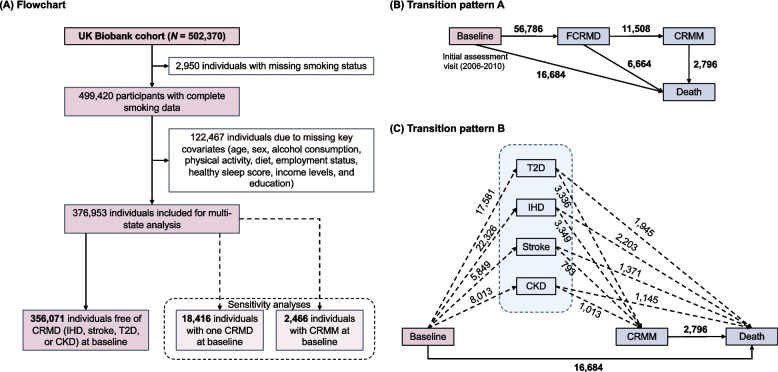
Flowchart and transition patterns of cardio-renal-metabolic multimorbidity in a prospective cohort study from the UK Biobank (baseline 2006–2010; follow-up through 2022). **A** Flowchart illustrating the inclusion and exclusion criteria for individuals in the multi-state analysis. **B** Number of participants across transitions pathways based on four states in CRMM progression (Transition pattern A). **C** Number of participants across transitions pathways based on seven states in CRMM progression. (Transition pattern B). Note that individuals who entered multiple CRM diseases on the same date were excluded, as the specific subtype of first CRM disease (FCRMD) could not be determined. Abbreviations: *N*, sample size; IHD, ischemic heart disease; T2D, type 2 diabetes; CKD, chronic kidney disease; FCRMD, first cardio-renal-metabolic disease; CRMM, cardio-renal-metabolic multimorbidity

Data used in this study were accessed via application *98273*. Of the 502,370 participants, we excluded those with missing smoking status (*N* = 2,950), missing key demographics or lifestyle covariates (i.e., age, sex, alcohol consumption, physical activity, diet, employment status, healthy sleep score, income levels, and education) (*N* = 122,467), or baseline ischemic heart disease (IHD), stroke, T2D, or CKD (*N* = 20,882), leaving 356,071 participants for subsequent analysis (Fig. 1(A)).

### Smoking behaviors

The exposures were smoking status (never, former and current) for all participants as well as years since quitting for former smokers. Three additional smoking-related traits, including smoking initiation age, smoking intensity, and years of smoking before quitting, were also considered in sensitivity and subgroup analyses. All these traits were assessed with questions from the touchscreen questionnaire on smoking lifestyle during the initial assessment visit (2006–2010). Specifically, all participants were classified as current, former and never smokers based on two questions: “*Do you smoke tobacco now?*” and “*In the past, how often have you smoked tobacco?*” (Field ID 20116). For all former and current smokers, smoking intensity was measured using the variable “Pack years of smoking” (Field ID 20161), with heavy smoking defined as smoking more than 20 pack-years, consistent with previous study [20]. For those individuals who had stopped smoking, self-reported data on smoking initiation age (Field ID 2867) and cessation age (Field ID 2897 and 6194) were used to calculate the years of smoking before quitting. The years since quitting were determined as the difference between baseline age and the age at which smoking cessation occurred. Additional details were available in Supplemental Table S1.

### CRM diseases and transition patterns of CRM multimorbidity

We considered four CRM diseases, including IHD, stroke, T2D, and CKD, in accordance with previous literature [6, 7]. They were defined respectively using the following International Statistical Classification of Diseases, Tenth Revision (ICD-10) diagnosis codes: (1) IHD: I20–I25; (2) stroke: I60–I69; (3) T2D: E11; and (4) CKD: N18 [26, 27], based on diagnoses recorded during hospital inpatient admissions (Field ID 41270). The corresponding dates of outcome diagnoses were extracted from Field ID 41280. We verified the vital and dropout status of each participant. For each outcome, follow-up time was calculated from the date of enrollment to the latest date among the date of first diagnosis, death (Field ID 40000), loss to follow-up (Field ID 191), and censoring date (defined as October 31, 2022, the latest diagnosis date for ICD 10 in Field ID 41280).

Our main analysis considered CRMM progression including four states and five transition pathways (transition pattern A, Fig. 1(B)): (1) baseline free of all four CRM diseases to FCRMD; (2) FCRMD to CRMM; (3) CRMM to all-cause death; (4) baseline to death from disease other than CRM diseases; and (5) FCRMD to all-cause death. The FCRMD was defined as the initial occurrence of any of IHD, stroke, T2D, or CKD; CRMM was defined as the incidence of a second CRMD. Death was defined using Field ID 40001. The date for each state was calculated as the date of entry into this status, death, loss to follow-up, or censoring (October 31, 2022), whichever occurred first. For participants entering multiple states (e.g., FCRMD and CRMM) on the same date, 0.5 days were subtracted from the entry date of the earlier state or added to that of the later state [6]. Finally, the entry and exit times for each transition pathway were derived using the *mstate* package in R to construct the multi-state transition dataset.

To further consider the potential differential associations of smoking behavior with CRMM progression according to subtypes of FCRMD, we split the CRMM progression by subtypes of FCRMD and constructed a transition pattern comprising 14 pathways based on seven states (transition pattern B, Fig. 1(C)), defined as baseline to one of the specific CRM diseases, then to CRMM, and subsequently to death. Specifically, in this part of the analysis, we additionally excluded individuals who entered multiple CRM diseases on the same date, as the specific subtype of FCRMD could not be determined, leaving 353,054 individuals for inclusion.

### Covariates

We predefined a set of baseline covariates for adjustment prior to the statistical analysis in order to fully consider the potential confounders between smoking traits and CRMM transition pathways, with reference to previous research on lifestyle factors and CRMM [5, 6]. The covariates included age, sex, alcohol consumption (never and consumer), physical activity (low, moderate, and high) [28, 29], diet (total fruit and vegetable intake < 5 and ≥ 5 portions/day) [28], employment status (working, unemployed, retired, and other) [28], healthy sleep score (0 to 5) [30], income levels (level 1 to 4), and education (< 10 years and ≥ 10 years) [31]. Additional covariates including body mass index (BMI), systolic blood pressure (SBP), total cholesterol (TC), the use of antihypertensive and statin/other cholesterol-lowering medications were further adjusted in the sensitivity analysis. Details were provided in Supplementary Method 1 and Table S2.

### Statistical analysis

Baseline characteristics were summarized by smoking status and compared using ANOVA/Kruskal–Wallis tests for continuous variables and χ^2^ tests for categorical variables. In the initial analyses, we used Cox proportional hazards models to estimate the associations between smoking behaviors (never smokers, former smokers with ≥ 25, 15–24, 10–14, 5–9, and < 5 years since quitting, and current smokers) and a range of outcomes. These included individual CRM diseases—CVD (defined as a composite of IHD and stroke), T2D, and CKD—as well as their combinations (CVD + T2D, T2D + CKD, CVD + CKD, and coexistence of all three diseases). We also evaluated different states in CRMM progression, including FCRMD, CRMM (≥ 2 CRM diseases), all-cause mortality, and cause-specific mortality (cardiovascular and non-cardiovascular), adjusting for all covariates. The proportional hazards assumption was assessed and verified based on Schoenfeld residuals. The effect sizes were expressed as hazard ratios (HRs) with 95% confidence intervals (CIs), calculated for each smoking category compared with never smokers, to determine the duration required for each single state in former smokers to reach a level that is not significantly different from that of never smokers. Additionally, the risk for each single state was compared with that of current smokers to assess the years needed for smoking cessation to lead to a significant risk reduction.

Then in the main analyses, multi-state modeling [32, 33] was used to comprehensively assess the role of smoking behavior in CRMM progression and prognosis, i.e., from baseline (free of CRM disease) to FCRMD, CRMM, and ultimately to death. Based on transition pattern A including four states and five transition pathways, the effect of smoking status and smoking cessation by years since quitting on different stages of CRMM progression was estimated simultaneously, with consideration of competing risks. The effects were then calculated based on the pattern dividing the FCRMD into four individual diseases (transition pattern B) to further clarify whether the influence of smoking varies according to pathways through different FCRMDs.

We further conducted sensitivity and subgroup analyses to evaluate the robustness of our findings. First, the pattern and magnitude of risk reduction for each single state across different durations since smoking cessation were explored using Cox models with restricted cubic splines (five knots) [20, 21] (see Supplementary Method 2 for details). Second, we further adjusted for SBP, TC, and the use of antihypertensive and lipid-lowering medications in multi-state models to evaluate the robustness of the main analysis results. Third, main analyses were repeated using the multiple imputation datasets (*N* = 469,945) to assess the robustness of the results to the handling of missing data. Fourth, to address potential bias arising from the time lag between the cohort baseline and the onset of later disease states in transition pattern A, we directly selected individuals who were already in the FCRMD (*N* = 18,416) or CRMM (*N* = 2,466) state at baseline (Fig. 1(A)). These participants were then followed for subsequent transitions (e.g., CRMM to death). Multivariable Cox proportional hazards models were applied to re-examine the associations of smoking status and years since cessation with each specific transition. Fifth, for transition pattern A, we examined the association between years of smoking prior to cessation (< 5, 5–9, 10–14, 15–24, and ≥ 25 years before quitting) and the risk of each transition among former smokers. Sixth, years since cessation was recalculated dynamically according to the start time of each transition state in the multi-state model to explore robustness under a time-dependent exposure setting. Finally, subgroup analyses were performed according to sex (male and female), age at smoking initiation (< 18 vs ≥ 18 years), smoking intensity (< 20 vs ≥ 20 pack-years), and genetic risk score (GRS) [30] (high, intermediate, or low; details were provided in Supplementary Method 2 and Table S3) for each individual FCRMD [34–37]. We evaluated multiplicative interactions using a likelihood ratio test, comparing models with and without the cross-product term, with both smoking behavior and the grouping variable treated as categorical.

All statistical analyses were conducted using R software (version 4.4.1). Statistical significance was defined as a two-sided *P* < 0.05. This study adhered to the Strengthening the Reporting of Observational Studies in Epidemiology (STROBE) guidelines throughout the analysis and reporting process.

## Results

### Study population and sample characteristics

This study included a total of 356,071 individuals, with a median age of 57 years (IQR: 49–62), of whom 44.9% were male. Among them, 55.1% were never smokers, 34.4% former smokers, and 10.5% current smokers (Table 1). Compared with never smokers, current smokers were more likely to be male and to have lower education and household income levels. Current smokers also exhibited a higher prevalence of unhealthy lifestyle factors, including lower vegetable intake, lower physical activity levels, and poorer sleep status. Among former smokers, the median time since quitting was 19 years (IQR: 8–28 years), and the median duration of smoking was 20 years (IQR: 12–30 years). Heavy smokers (cumulative smoking amount ≥ 20 pack-years) accounted for 40% of both former and current smokers.

**Table 1:** Baseline characteristics of participants according to smoking status in a prospective cohort study from the UK Biobank (*N* = 356,071; baseline 2006–2010; follow-up through 2022)

Variable	Never Smokers (*N* = 196,131)	Former Smokers (*N* = 122,359)	Current Smokers (*N* = 37,581)	*P* value
Sex, No. (%)				
Female	116,153 (59.2)	62,369 (51.0)	17,826 (47.4)	< 0.001
Male	79,978 (40.8)	59,990 (49.0)	19,755 (52.6)	
Age, years, median [IQR]	56.0 [48.0, 62.0]	59.0 [52.0, 64.0]	54.0 [47.0, 61.0]	< 0.001
Alcohol consumption, No. (%)				
Never	15,987 (8.2)	6061 (5.0)	2521 (6.7)	< 0.001
Drinker	180,144 (91.8)	116,298 (95.0)	35,060 (93.3)	
Physical activity, No. (%)				
Low	45,905 (23.4)	27,855 (22.8)	10,557 (28.1)	< 0.001
Moderate	97,022 (49.5)	59,545 (48.7)	16,155 (43.0)	
High	53,204 (27.1)	34,959 (28.6)	10,869 (28.9)	
Fruit and vegetable intake, No. (%)				
< 5 portions/day	151,569 (77.3)	93,880 (76.7)	32,514 (86.5)	< 0.001
≥ 5 portions/day	44,562 (22.7)	28,479 (23.3)	5067 (13.5)	
Employment status, No. (%)				
Working	127,944 (65.2)	71,204 (58.2)	24,190 (64.4)	< 0.001
Unemployed	2475 (1.3)	1556 (1.3)	1273 (3.4)	
Retired	55,480 (28.3)	43,352 (35.4)	8277 (22.0)	
Other	10,232 (5.2)	6247 (5.1)	3841 (10.2)	
Healthy sleep score, No. (%)				
0	358 (0.2)	300 (0.2)	187 (0.5)	< 0.001
1	7417 (3.8)	6196 (5.1)	2854 (7.6)	
2	36,643 (18.7)	26,848 (21.9)	10,700 (28.5)	
3	74,199 (37.8)	47,745 (39.0)	14,069 (37.4)	
4	61,837 (31.5)	34,145 (27.9)	8078 (21.5)	
5	15,677 (8.0)	7125 (5.8)	1693 (4.5)	
Household income, No. (%)				
Level 1	36,688 (18.7)	27,059 (22.1)	12,075 (32.1)	< 0.001
Level 2	47,952 (24.4)	32,306 (26.4)	9686 (25.8)	
Level 3	53,868 (27.5)	32,136 (26.3)	8771 (23.3)	
Level 4	57,623 (29.4)	30,858 (25.2)	7049 (18.8)	
Education level, No. (%)				
< 10 years	53,929 (27.5)	40,467 (33.1)	14,878 (39.6)	< 0.001
≥ 10 years	142,202 (72.5)	81,892 (66.9)	22,703 (60.4)	
SBP, mmHg, median [IQR]	135.0 [123.5, 148.0]	137.5 [125.5, 151.0]	133.0 [122.0, 146.5]	< 0.001
TC, mmol/L, median [IQR]	5.7 [5.0, 6.5]	5.7 [5.0, 6.5]	5.7 [5.0, 6.5]	0.027
BMI, kg/m^2^, median [IQR]	26.3 [23.8, 29.4]	27.1 [24.5, 30.2]	26.3 [23.7, 29.4]	< 0.001
Antihypertensive medication, No. (%)				
No	162,547 (82.9)	95,460 (78.0)	31,647 (84.2)	< 0.001
Yes	33,584 (17.1)	26,899 (22.0)	5934 (15.8)	
Statin use, No. (%)				
No	173,916 (88.7)	101,700 (83.1)	32,249 (85.8)	< 0.001
Yes	22,215 (11.3)	20,659 (16.9)	5332 (14.2)	

Continuous variables were presented as mean (SD) or median (IQR), based on the result of normality assessment using the Shapiro–Wilk test. Categorical variables were presented as No. (%)

*Abbreviations*: *N* sample size, *P* *P* value, *No*. number of subjects, *%* percentage, *SD* Standard deviation, *IQR* Interquartile range, *SBP* Systolic blood pressure, *TC* Total cholesterol, *BMI* Body mass index

During a median follow-up of 13.6 years (IQR: 12.7–14.3 years), 56,786 participants experienced FCRMD. Among them, the FCRMDs were T2D in 17,581 individuals, IHD in 22,326, stroke in 5,849, and CKD in 8,013. Among all participants who developed a first CRM disease, 11,508 progressed to CRMM, of whom 2,796 subsequently died. A total of 16,684 participants died without experiencing any CRM disease, and 6,664 died directly after their first CRM disease.

### Cox regression analyses of smoking behaviors and CRM outcomes

**Fig. 2:**
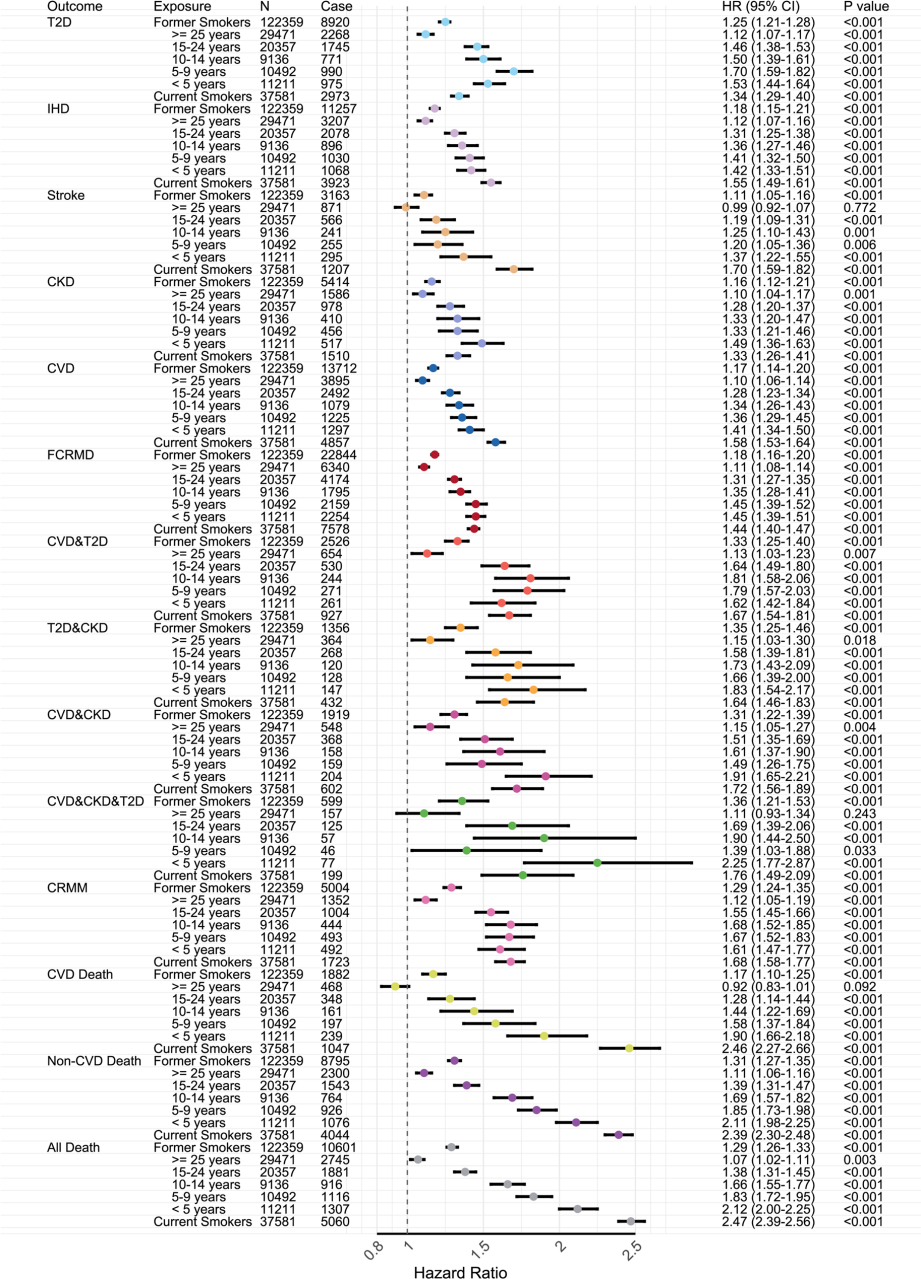
Association of smoking status and year since quitting with individual cardio-renal-metabolic diseases and cardio-renal-metabolic multimorbidity states in a prospective cohort study from the UK Biobank (baseline 2006–2010; follow-up through 2022). Cox proportional hazards models adjusted for age, sex, alcohol consumption, physical activity, diet, employment status, healthy sleep score, income levels, and education. Abbreviations: *N*, total sample size; Case, number of individuals experience the transition; *HR*, hazard ratio; *CI*, confidence interval; *P*, *P* value; CRM, cardio-renal-metabolic; FCRMD, first cardio-renal-metabolic disease; CRMM, cardio-renal-metabolic multimorbidity

Compared with never smokers, current smokers were associated with a 1.34-fold higher risk of T2D (95% *CI*: 1.29–1.40), a 1.55-fold higher risk of IHD (95% *CI*: 1.49–1.61), a 1.70-fold higher risk of stroke (95% *CI*: 1.59–1.82), and a 1.33-fold higher risk of CKD (95% *CI*: 1.26–1.41) (Fig. 2). They also had a 44% higher risk of FCRMD (*HR* = 1.44; 95% *CI*: 1.40–1.47), a 68% higher risk of CRMM (*HR* = 1.68; 95% *CI*: 1.58–1.77), and a 147% higher risk of all-cause death (*HR* = 2.47; 95% *CI*: 2.39–2.56).

More than 25 years were generally needed for the risk of FCRMD, CRMM, or all-cause death to decline to levels that were not statistically different from those in never smokers, with corresponding HRs (95% CIs) of 1.11 (1.08–1.14), 1.12 (1.05–1.19), and 1.07 (1.02–1.11) for those quitting ≥ 25 years (Fig. 2, Supplementary Figure S1-S2). Specifically, the risks of stroke and CVD death were no longer significantly different from those of never smokers after approximately 20 years of cessation, with HRs (95% CIs) of 0.99 (0.92–1.07) and 0.92 (0.83–1.01), respectively, in the ≥ 25-year cessation group. Compared to current smokers, the risk reduction associated with duration of smoking cessation varied for different CRMM states. A significant protective effect was observed approximately 7 years after cessation for FCRMD (*HR* = 0.93; 95% *CI*: 0.88–0.98 in the 10–14-year cessation group) and 22 years for CRMM (*HR* = 0.92; 95% *CI*: 0.85–0.99 in the 15–24-year cessation group), whereas for all-cause mortality, it became evident almost immediately after quitting (*HR* = 0.85; 95% *CI*: 0.80–0.90 in the < 5-year cessation group). The temporal patterns of risk reduction across individual CRM diseases relative to current smokers also appeared to differ. The risk for IHD (*HR* = 0.91; 95% *CI*: 0.85–0.98), stroke (*HR* = 0.80; 95% *CI*: 0.71–0.91), and CVD (*HR* = 0.89; 95% *CI*: 0.84–0.94) decreased significantly within the first 5 years after cessation, whereas those for T2D and CKD increased shortly after cessation before significant protective effects became evident after around 20 years and 23 years, respectively.

### Association of smoking behaviors with transitions in CRMM progression

**Fig. 3:**
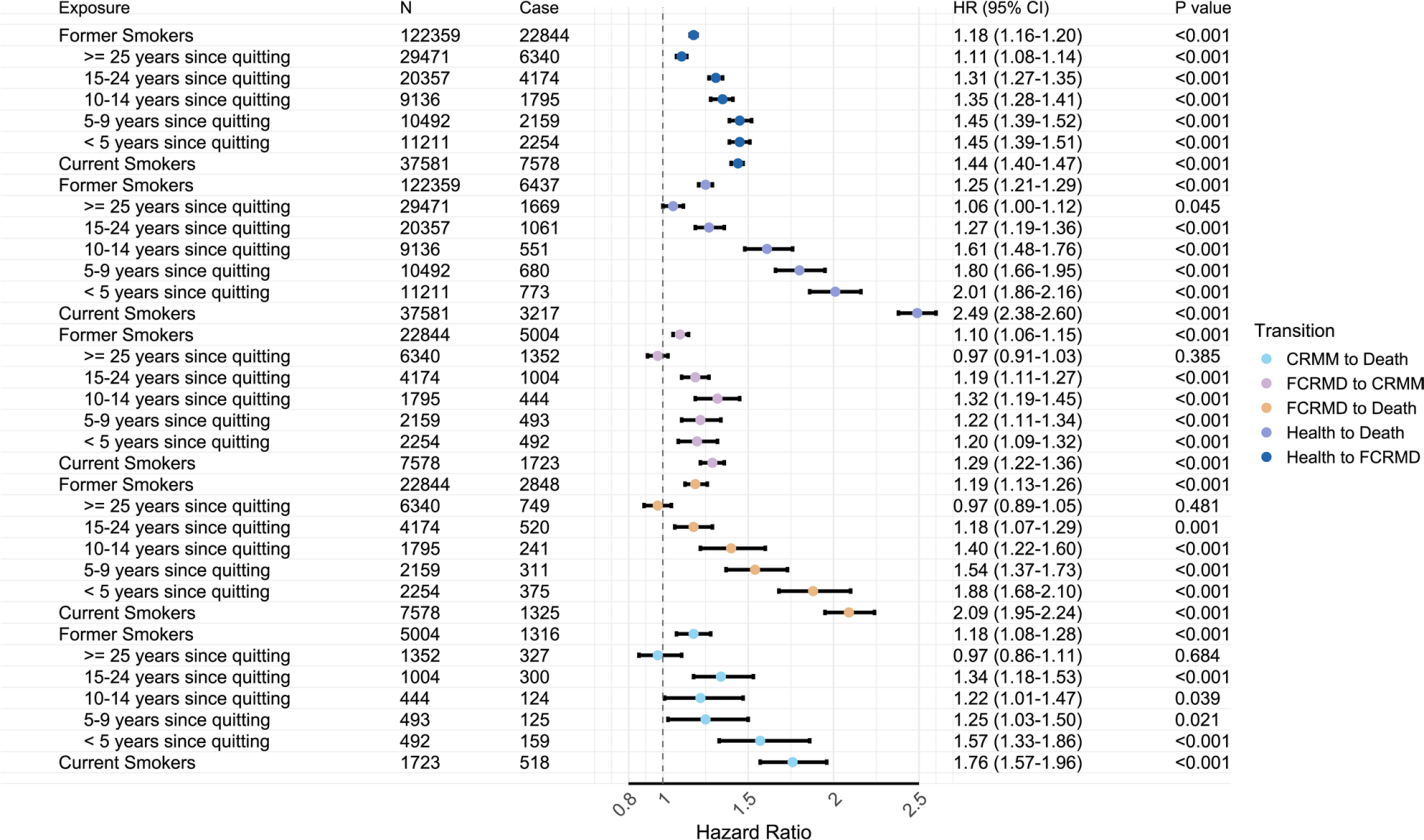
Association of smoking status and year since quitting with pathways in cardio-renal-metabolic multimorbidity transition pattern A in a prospective cohort study from the UK Biobank (baseline 2006–2010; follow-up through 2022). Multi-state models were used to assess the role of smoking status in temporal disease progression from baseline (free of CRM disease) to FCRMD, CRMM, and ultimately to death. All models were adjusted for age, sex, alcohol consumption, physical activity, diet, employment status, healthy sleep score, income levels, and education. Transition pattern A: transition pathways based on four states in CRMM progression (see Fig. 1B). Abbreviations: *N*, total sample size; Case, number of individuals experience the transition; *HR*, hazard ratio; *CI*, confidence interval; *P*, *P* value; CRM, cardio-renal-metabolic; FCRMD, first cardio-renal-metabolic disease; CRMM, cardio-renal-metabolic multimorbidity

Multi-state models further evaluated the association between baseline smoking behavior and risk of all transitions from a healthy state (free of FCRMD) to FCRMD, then to CRMM, and ultimately to all-cause mortality with increasing years since smoking cessation (Fig. 3). Across all five progression transitions, current smoking had a greater impact on the transition from different states to mortality compared with never smokers. The HRs for transitions to death were 2.49 (95% *CI*: 2.38–2.60) from a healthy state, 2.09 (95% *CI*: 1.95–2.24) from FCRMD, and 1.76 (95% *CI*: 1.57–1.96) from CRMM, whereas the HR for the transition from a healthy state to FCRMD was comparatively lower at 1.44 (95% *CI*: 1.40–1.47) and was further attenuated for the transition from FCRMD to CRMM (*HR* = 1.29, 95% *CI*: 1.22–1.36).

**Fig. 4:**
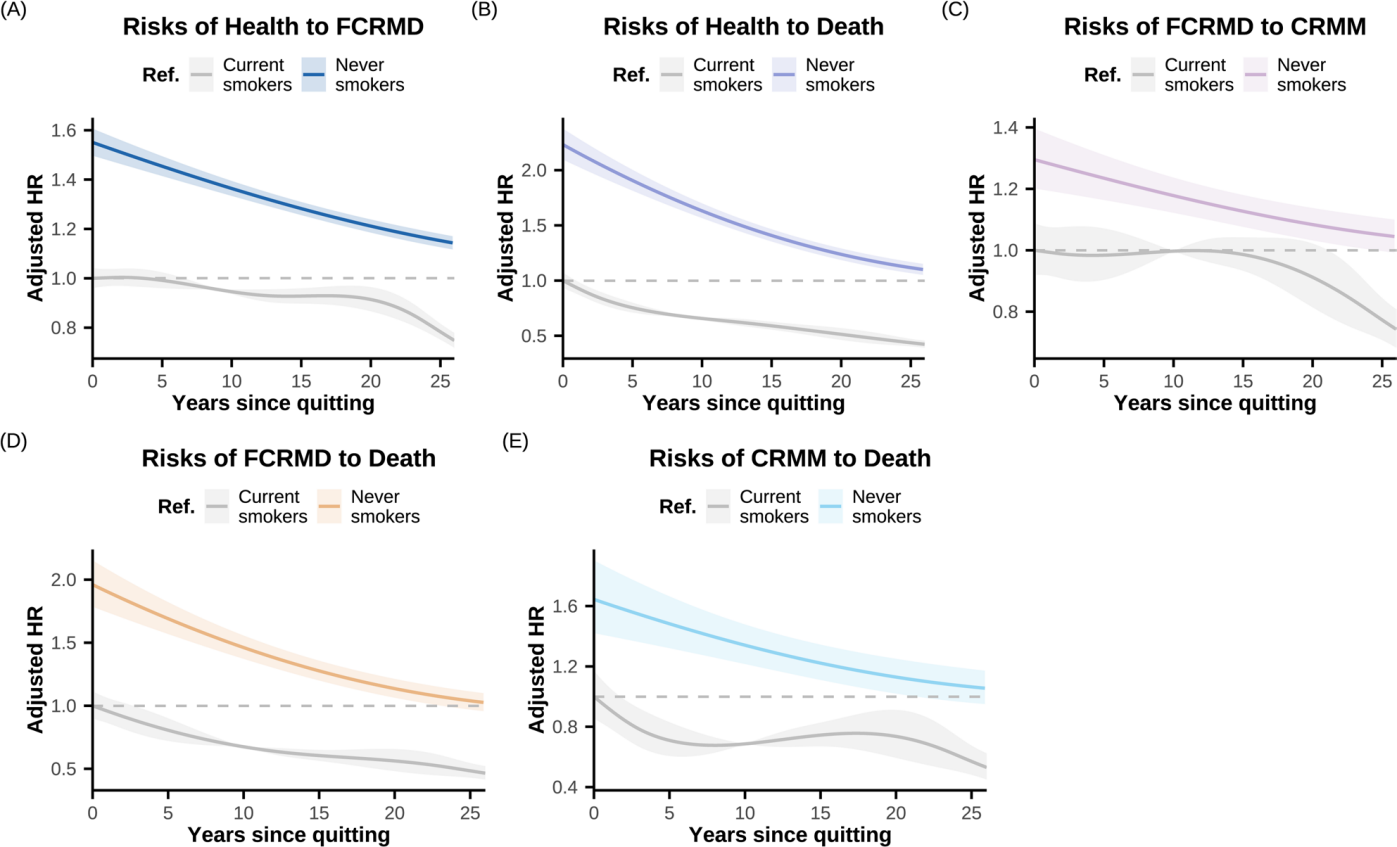
Risk for pathways in the cardio-renal-metabolic multimorbidity transition pattern A by years since quitting in a prospective cohort study from the UK Biobank (baseline 2006–2010; follow-up through 2022). Restricted cubic splines with five knots were employed to capture potential nonlinear associations between years since quitting and the log hazard of each transition pathway. Years since quitting were treated as a continuous variable, with durations exceeding 25 years set to 26 (range: 0–26). Two analyses were performed: (1) comparisons of former and current smokers (assigning a value of 0 for years since quitting) with never smokers (for whom years since quitting was set to 50, significantly larger than that of all former smokers, following the approach used in previous studies), shown as colored lines; and (2) comparisons of former smokers with current smokers, shown as gray lines. All models were adjusted for age, sex, alcohol consumption, physical activity, diet, employment status, healthy sleep score, income levels, and education. Abbreviations: Ref. reference group; *HR*, hazard ratio; FCRMD, first cardio-renal-metabolic disease; CRMM, cardio-renal-metabolic multimorbidity. Transition pattern A, transitions pathways based on four states in CRMM progression (see Fig. 1B)

Former smokers reached risk levels that were not statistically different from those of never-smokers for all transitions in CRMM progression after approximately 25 years of cessation (Figs. 3 and 4). Compared with current smokers (Fig. 4 and Supplementary Figure S3), smoking cessation was associated with a rapid, significant reduction (within 5 years) in mortality risks among individuals with a healthy state (*HR* = 0.80, 95% *CI*: 0.74–0.87) or FCRMD (*HR* = 0.89, 95% *CI*: 0.79–0.99), with gradual declines over 25 years. For the transition from CRMM to death, a modest short-term reduction was followed by a plateau (5–20 years) and further decline thereafter. Significant risk reductions for the transitions from a health state to FCRMD (*HR* = 1.00, 95% *CI*: 0.96–1.05) and from FCRMD to CRMM (*HR* = 0.93, 95% *CI*: 0.84–1.03) were not observed in the short term; for the latter transition, a significant risk decrease only observed after more than 20 years of cessation. Sensitivity analyses adjusting for additional covariates, using imputed dataset, incorporating dynamically updated years since cessation, and using baseline FCRMD or CRMM populations yielded broadly consistent results (Supplementary Table S4, Figure S4-S7).

### Association of smoking behaviors with disease-specific transitions in CRMM progression

**Fig. 5:**
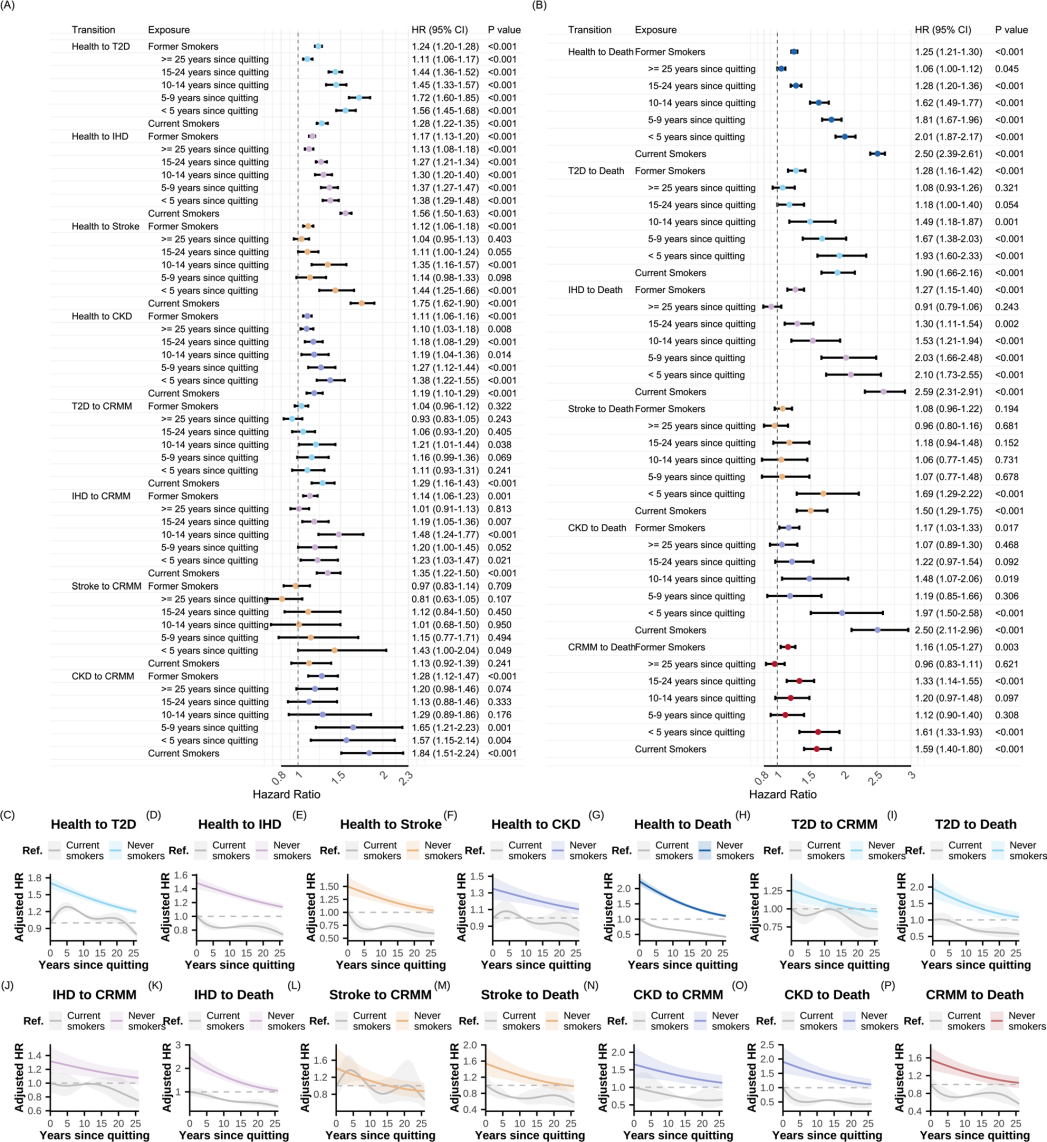
Association of smoking status and year since quitting with pathways in the cardio-renal-metabolic multimorbidity transition pattern B in a prospective cohort study from the UK Biobank (baseline 2006–2010; follow-up through 2022). **A** Multi-state model was used to assess the role of smoking status in the temporal disease progression from baseline (free of CRM disease) to each individual FCRMD, CRMM, and ultimately to death. **B**-**P** Restricted cubic splines with five knots were employed to capture potential nonlinear associations between years since quitting and the log hazard of each transition pathway. All models were adjusted for age, sex, alcohol consumption, physical activity, diet, employment status, healthy sleep score, income levels, and education. Abbreviations: *HR*, hazard ratio; *CI*, confidence interval; *P*, *P* value; Ref. reference group; IHD, ischemic heart disease; T2D, type 2 diabetes; CKD, chronic kidney disease; CRM, cardio-renal-metabolic; FCRMD, first cardio-renal-metabolic disease; CRMM, cardio-renal-metabolic multimorbidity. Transition pattern B, transitions pathways based on seven states in CRMM progression (see Fig. 1C)

As shown in Fig. 5, for transitions from specific types of FCRMD to CRMM, current smokers had elevated risks for the transitions of three FCRMDs: *HR* = 1.29 (95% *CI*: 1.16–1.43) for T2D, 1.35 (95% *CI*: 1.22–1.50) for IHD, and 1.84 (95% *CI*: 1.51–2.24) for CKD, but not stroke (*HR* = 1.13, 95% *CI*: 0.92–1.39). For transitions from IHD or CKD to CRMM, approximately 25 years of smoking cessation are required for the risk levels of former smokers to become statistically indistinguishable from those of never smokers, whereas around 15 years are required for the transition from T2D. Compared with current smokers, former smokers have a significant decreased risk for transitions from CKD after around 10 years, whereas more than 20 years are required for transitions from IHD and T2D.

For the transitions from individual FCRMD to death, the effects of current smoking were largest for transitions from IHD (*HR* = 2.59, 95% *CI*: 2.31–2.91), followed by CKD (*HR* = 2.50, 95% *CI*: 2.11–2.96), T2D (*HR* = 1.90, 95% *CI*: 1.66–2.16), and stroke (*HR* = 1.50, 95% *CI*: 1.29–1.75). After approximately 25 years of abstinence, the risk levels among former smokers of transitions from IHD, CKD, and T2D became not significantly different from those of never smokers, whereas around 15 years are required for the transition from stroke. Notably, only the CKD- and stroke-to-death transitions demonstrated significant risk decreases soon after smoking cessation (within 5 years) compared with current smokers (Fig. 5 and Supplementary Figure S8).

### Sensitivity and subgroup analyses

Significant multiplicative interactions were observed between smoking behaviors and sex or age at smoking initiation for certain transitions in CRMM progression (Table 2). For the transitions from healthy to FCRMD, FCRMD to CRMM, and CRMM to death, risks were significantly higher in female current smokers than in male current smokers. Nevertheless, compared with male quitters, female quitters exhibited significant risk reductions relative to current smokers within a much shorter duration of cessation (e.g., 5 years for the health-to-FCRMD transition in females vs. 25 years in males). Similar patterns were observed among individuals who began smoking before age 18 compared with those who initiated smoking in adulthood. For the transitions from health or FCRMD to death, early current smokers had significantly higher risks relative to never smokers, but upon quitting, former smokers experienced greater risk reductions over the same cessation duration compared with current smokers.

**Table 2:** Associations of smoking status and years since quitting with progression of cardio-renal-metabolic multimorbidity (transition pattern A) and interactions with sex and age at smoking initiation in a prospective cohort study from the UK Biobank (baseline 2006–2010; follow-up through 2022)

	**Analysis (a): never smokers as reference**				**Analysis (b): Current smokers as reference**			
	**Sex**		**Age at initiation**		**Sex**		**Age at initiation**	
	**Female**	**Male**	** < 18 years old**	** ≥18 years old**	**Female**	**Male**	** < 18 years old**	** ≥18 years old**
	***HR* (95% *CI*)**	***HR* (95% *CI*)**	***HR* (95% *CI*)**	***HR* (95% *CI*)**	***HR* (95% *CI*)**	***HR* (95% *CI*)**	***HR* (95% *CI*)**	***HR* (95% *CI*)**
Health to FCRMD								
Never smokers	1.00	1.00	1.00	1.00	0.65(0.62,0.68)	0.72(0.70,0.75)	0.63(0.61,0.65)	0.71(0.69,0.74)
Former smokers	1.12(1.09,1.15)	1.23(1.20,1.26)	1.32(1.29,1.35)	1.18(1.15,1.22)	0.73(0.70,0.76)	0.89(0.86,0.92)	0.83(0.80,0.86)	0.85(0.81,0.89)
≥25 years since quitting	1.05(1.00,1.10)	1.15(1.11,1.19)	1.15(1.11,1.19)	1.05(1.01,1.10)	0.68(0.64,0.72)	0.82(0.79,0.86)	0.72(0.69,0.75)	0.75(0.71,0.79)
15–24 years since quitting	1.22(1.15,1.29)	1.38(1.32,1.43)	1.39(1.34,1.45)	1.21(1.15,1.27)	0.79(0.74,0.84)	0.99(0.94,1.03)	0.87(0.83,0.92)	0.86(0.81,0.92)
10–14 years since quitting	1.28(1.18,1.39)	1.40(1.31,1.48)	1.40(1.32,1.49)	1.28(1.19,1.38)	0.83(0.76,0.90)	1.00(0.94,1.07)	0.88(0.82,0.94)	0.91(0.84,0.99)
5–9 years since quitting	1.40(1.30,1.50)	1.49(1.41,1.58)	1.55(1.47,1.64)	1.32(1.23,1.41)	0.90(0.84,0.98)	1.07(1.01,1.14)	0.97(0.91,1.03)	0.94(0.87,1.02)
< 5 years since quitting	1.50(1.40,1.61)	1.42(1.34,1.50)	1.51(1.43,1.60)	1.36(1.27,1.46)	0.97(0.90,1.05)	1.02(0.96,1.08)	0.95(0.89,1.01)	0.97(0.90,1.05)
Current Smokers	1.54(1.47,1.60)	1.39(1.34,1.43)	1.59(1.54,1.65)	1.40(1.34,1.46)	1.00	1.00	1.00	1.00
	*P* for interaction (Sex): Method 1 = 6.51 × 10^–12^; Method 2 = 1.99 × 10^–8^				*P* for interaction (Age): Method 1 = 0.45; Method 2 = 0.59			
Health to Death								
Never smokers	1.00	1.00	1.00	1.00	0.38(0.36,0.41)	0.43(0.40,0.45)	0.34(0.32,0.36)	0.41(0.38,0.43)
Former smokers	1.30(1.23,1.36)	1.20(1.14,1.26)	1.38(1.32,1.44)	1.31(1.25,1.38)	0.49(0.46,0.53)	0.51(0.48,0.54)	0.47(0.44,0.49)	0.54(0.50,0.58)
≥25 years since quitting	1.10(1.01,1.20)	1.01(0.94,1.09)	1.06(0.99,1.13)	1.06(0.98,1.15)	0.41(0.38,0.46)	0.43(0.40,0.46)	0.35(0.33,0.38)	0.43(0.39,0.47)
15–24 years since quitting	1.33(1.21,1.47)	1.21(1.11,1.32)	1.31(1.20,1.42)	1.24(1.12,1.36)	0.50(0.45,0.56)	0.51(0.47,0.56)	0.44(0.40,0.48)	0.50(0.45,0.56)
10–14 years since quitting	1.64(1.43,1.88)	1.57(1.40,1.75)	1.64(1.47,1.84)	1.60(1.40,1.82)	0.62(0.54,0.72)	0.66(0.59,0.74)	0.55(0.49,0.62)	0.65(0.56,0.75)
5–9 years since quitting	1.85(1.64,2.09)	1.75(1.57,1.94)	1.91(1.73,2.12)	1.67(1.48,1.89)	0.70(0.61,0.79)	0.74(0.66,0.82)	0.64(0.57,0.71)	0.68(0.59,0.77)
< 5 years since quitting	2.14(1.91,2.39)	1.89(1.71,2.09)	2.13(1.94,2.35)	1.86(1.66,2.09)	0.81(0.72,0.91)	0.80(0.72,0.88)	0.71(0.64,0.79)	0.75(0.66,0.86)
Current Smokers	2.62(2.45,2.79)	2.35(2.22,2.49)	2.96(2.80,3.13)	2.46(2.30,2.62)	1.00	1.00	1.00	1.00
	*P* for interaction (Sex): Method 1 = 0.14; Method 2 = 0.62				*P* for interaction (Age): Method 1 = 3.30 × 10^–3^; Method 2 = 0.04			
FCRMD to CRMM								
Never smokers	1.00	1.00	1.00	1.00	0.70(0.64,0.76)	0.82(0.76,0.88)	0.78(0.72,0.84)	0.76(0.70,0.83)
Former smokers	1.07(1.00,1.14)	1.12(1.06,1.18)	1.14(1.09,1.20)	1.08(1.02,1.15)	0.75(0.68,0.82)	0.92(0.86,0.99)	0.89(0.82,0.96)	0.83(0.75,0.91)
≥25 years since quitting	0.97(0.87,1.09)	0.97(0.90,1.05)	1.00(0.93,1.08)	0.93(0.85,1.03)	0.68(0.60,0.78)	0.80(0.73,0.87)	0.78(0.71,0.85)	0.71(0.63,0.81)
15–24 years since quitting	1.16(1.03,1.32)	1.20(1.10,1.30)	1.23(1.13,1.33)	1.12(1.01,1.25)	0.81(0.71,0.94)	0.98(0.89,1.08)	0.95(0.86,1.06)	0.86(0.75,0.97)
10–14 years since quitting	1.21(1.01,1.45)	1.36(1.21,1.53)	1.33(1.18,1.50)	1.28(1.10,1.50)	0.85(0.70,1.03)	1.11(0.98,1.26)	1.03(0.90,1.18)	0.97(0.82,1.16)
5–9 years since quitting	1.18(1.00,1.38)	1.24(1.10,1.39)	1.26(1.12,1.41)	1.16(1.00,1.35)	0.82(0.69,0.98)	1.01(0.89,1.14)	0.97(0.86,1.11)	0.88(0.75,1.05)
< 5 years since quitting	1.11(0.95,1.30)	1.25(1.11,1.40)	1.19(1.06,1.33)	1.22(1.05,1.42)	0.78(0.66,0.92)	1.02(0.90,1.15)	0.92(0.81,1.05)	0.93(0.79,1.10)
Current Smokers	1.43(1.31,1.57)	1.22(1.13,1.31)	1.28(1.19,1.38)	1.31(1.20,1.43)	1.00	1.00	1.00	1.00
	*P* for interaction (Sex): Method 1 = 8.15 × 10^–3^; Method 2 = 0.09				*P* for interaction (Age): Method 1 = 0.27; Method 2 = 0.79			
FCRMD to Death								
Never smokers	1.00	1.00	1.00	1.00	0.44(0.40,0.49)	0.50(0.46,0.55)	0.41(0.38,0.45)	0.52(0.47,0.58)
Former smokers	1.17(1.07,1.28)	1.19(1.11,1.28)	1.21(1.13,1.30)	1.25(1.16,1.35)	0.52(0.46,0.58)	0.60(0.55,0.65)	0.50(0.46,0.55)	0.65(0.58,0.73)
≥25 years since quitting	0.95(0.81,1.10)	0.98(0.88,1.08)	0.89(0.80,0.99)	1.09(0.97,1.23)	0.41(0.35,0.49)	0.48(0.43,0.54)	0.36(0.32,0.41)	0.57(0.49,0.66)
15–24 years since quitting	1.14(0.95,1.35)	1.19(1.06,1.34)	1.22(1.08,1.37)	1.12(0.97,1.29)	0.50(0.41,0.60)	0.59(0.52,0.66)	0.50(0.44,0.57)	0.58(0.49,0.68)
10–14 years since quitting	1.30(1.02,1.66)	1.44(1.23,1.69)	1.36(1.15,1.62)	1.45(1.19,1.77)	0.57(0.44,0.74)	0.71(0.60,0.83)	0.56(0.46,0.66)	0.75(0.60,0.94)
5–9 years since quitting	1.31(1.06,1.63)	1.66(1.44,1.91)	1.58(1.37,1.83)	1.48(1.23,1.78)	0.58(0.46,0.72)	0.82(0.70,0.95)	0.65(0.55,0.76)	0.77(0.62,0.94)
< 5 years since quitting	1.76(1.47,2.12)	1.96(1.71,2.24)	1.94(1.69,2.22)	1.76(1.48,2.10)	0.77(0.64,0.94)	0.96(0.83,1.11)	0.79(0.68,0.91)	0.91(0.75,1.11)
Current Smokers	2.26(2.02,2.52)	2.00(1.83,2.18)	2.43(2.23,2.64)	1.92(1.73,2.13)	1.00	1.00	1.00	1.00
	*P* for interaction (Sex): Method 1 = 0.34; Method 2 = 0.20				*P* for interaction (Age): Method 1 = 4.00 × 10^–4^; Method 2 = 5.60 × 10^–4^			
CRMM to Death								
Never smokers	1.00	1.00	1.00	1.00	0.47(0.40,0.56)	0.63(0.55,0.73)	0.52(0.46,0.60)	0.60(0.51,0.70)
Former smokers	1.13(0.98,1.31)	1.18(1.06,1.31)	1.23(1.11,1.37)	1.15(1.02,1.30)	0.53(0.44,0.64)	0.75(0.65,0.85)	0.64(0.56,0.74)	0.69(0.57,0.83)
≥25 years since quitting	1.01(0.78,1.31)	0.95(0.82,1.10)	1.00(0.86,1.16)	0.93(0.75,1.14)	0.47(0.36,0.63)	0.60(0.51,0.71)	0.52(0.43,0.62)	0.55(0.43,0.71)
15–24 years since quitting	1.27(0.99,1.64)	1.35(1.16,1.58)	1.39(1.19,1.64)	1.28(1.04,1.56)	0.60(0.45,0.79)	0.85(0.72,1.01)	0.72(0.60,0.87)	0.76(0.60,0.97)
10–14 years since quitting	0.94(0.63,1.40)	1.31(1.06,1.63)	1.21(0.96,1.53)	1.21(0.90,1.63)	0.44(0.29,0.66)	0.83(0.66,1.04)	0.63(0.49,0.81)	0.72(0.52,1.00)
5–9 years since quitting	1.15(0.80,1.65)	1.27(1.02,1.58)	1.28(1.02,1.61)	1.18(0.87,1.61)	0.54(0.37,0.78)	0.80(0.63,1.01)	0.66(0.52,0.85)	0.71(0.51,0.99)
< 5 years since quitting	1.36(0.99,1.86)	1.66(1.36,2.03)	1.63(1.33,2.00)	1.47(1.12,1.94)	0.64(0.46,0.89)	1.04(0.84,1.29)	0.85(0.68,1.06)	0.88(0.65,1.20)
Current Smokers	2.12(1.78,2.53)	1.58(1.37,1.82)	1.91(1.67,2.19)	1.67(1.42,1.97)	1.00	1.00	1.00	1.00
	*P* for interaction (Sex): Method 1 = 8.34 × 10^–3^; Method 2 = 0.03				*P* for interaction (Age): Method 1 = 0.60; Method 2 = 0.99			

Smoking-related hazard ratios were estimated within strata defined by sex or age at smoking initiation using multi-state models. Models stratified by sex were adjusted for age, alcohol consumption, physical activity, diet, employment status, healthy sleep score, income levels, and education, whereas models stratified by age at initiation were additionally adjusted for sex

*Abbreviations*: *HR*, Hazard ratio, *CI* Confidence interval, *Ref.*, Reference group, *FCRMD* First cardio-renal-metabolic disease, *CRMM*, Cardio-renal-metabolic multimorbidity. Transition pattern A, transitions pathways based on four states in CRMM progression (see Fig. 1B)

Method 1: *P* value for interaction between smoking status and sex or age at initiation on the multiplicative scale, evaluated using a likelihood ratio test comparing models with and without the cross-product term, with smoking status (never, former, current smoker) and sex (male, female) or age at initiation (< 18, ≥18 years old) treated as categorical variables

Method 2: *P* value for interaction between years since quitting and sex or age at initiation on the multiplicative scale, evaluated using a likelihood ratio test comparing models with and without the cross-product term, with years since quitting (< 5, 5–9, 10–14, 15–24, ≥25) and sex (male, female) or age at initiation (< 18, ≥18 years old) treated as categorical variables

The duration and intensity of smoking substantially influenced the effect of smoking cessation on various CRMM transitions. Former smokers with a smoking history of less than 15 years had no significant excess risk for any transition compared with never smokers (Supplementary Figure S9). However, those with 15–24 years still had higher risks for the transition from health to FCRMD (*HR* = 1.27, 95% *CI*: 1.23–1.31) and from health to death (*HR* = 1.16, 95% *CI*: 1.09–1.24), but both were lower than risks of current smokers. Light-intensity former smokers showed immediate benefit from smoking cessation for the transition from CRMM to death and no excess risk for FCRMD to CRMM. In contrast, heavy-intensity former smokers required substantially longer cessation to reach risk levels that were not significantly different from those of never smokers (Supplementary Figure S10-S14).

Within subgroups stratified by genetic risk for each individual FCRMD, cessation effects on health-to-FCRMD disease-specific transitions were generally consistent (Supplementary Figure S15), with no significant multiplicative interactions observed (Supplementary Table S5). Current smokers with a high GRS for T2D or IHD had substantially higher risks for the transitions from health to T2D and from health to IHD, respectively, compared with never smokers with a low GRS (Supplementary Figure S16). For example, the HR for current smokers with a high-weighted T2D GRS was 5.40 (95% *CI*: 4.87–5.99) for the transition from health to T2D, and the HR for those with a high-weighted IHD GRS was 2.89 (95% *CI*: 2.64–3.16) for the transition from health to IHD.

## Discussion

Using the large-scale UK Biobank cohort, we systematically assessed the effects of smoking status and years since smoking cessation on the initiation, progression, and prognosis of CRMM. Our results mainly include four key points. First, across all transitions in CRMM progression, current smokers exhibited greater risks for transitions leading to death. The HRs varies from 1.29 (95% *CI*: 1.22–1.36) for the transition from FCRMD to CRMM to 2.49 (95% *CI*: 2.38–2.60) for the transition from a healthy state (free of CRM disease) to death. Among disease-specific transitions, current smoking was significantly associated with increased risks of transitions from CKD, IHD, and T2D to CRMM. For the transitions from FCRMD to death, the risk in current smokers varied by CRM disease type, ranging from a 50% increase for stroke to a 159% increase for IHD. Second, more than 25 years were required for all CRMM progression transitions before risks for former smokers became non-significantly different from those of never smokers. The time period required for risk convergence in the transitions from T2D to CRMM and from stroke to death tended to be shorter (approximately 15 years). Third, compared with current smokers, short-term smoking cessation was associated with statistically significant reductions in risk for transitions leading to death, whereas reductions for the transitions from health to FCRMD and from FCRMD to CRMM required longer cessation durations to become evident (> 5 and 20 years, respectively). Special attention is needed in the early post-cessation period for elevated risks of transition from health to T2D or CKD, as well as from IHD or T2D to death. Fourth, current smoking had a greater impact among female and individuals who initiated smoking before the age of 18; nevertheless, smoking cessation in these subgroups led to a marked reduction in risk in short term compared with current smoking. The benefits of smoking cessation were influenced by previous smoking behavior but not by genetic susceptibility to individual CRM diseases.

Regarding the impact of smoking cessation on the progression from the health stage to individual CRM diseases, the reduction trend in the risk of progression from the health stage to IHD and stroke was consistent with previous research. For example, a cohort study from the Framingham Heart Study found that CVD incidence fell significantly within five years of quitting, and the risk was no longer significantly higher than that of never smokers after 10–15 years [20]. Meanwhile, our finding that the risk-reducing effect of smoking cessation is influenced by cumulative smoking exposure is consistent with a Korean cohort study. That study found that former heavy smokers (> 8 pack-years) required more than 25 years for CVD risk to approach that of never smokers, substantially longer than former light smokers [21]. Regarding the differences in the patterns of risk reduction for CHD and stroke following smoking cessation, our previous UK Biobank analysis in participants with CKM stages 0–3 showed longer cessation was needed for cardiovascular diseases (> 25 years) than for cerebrovascular disease (around 15 years) [38]. This is consistent with the shorter time to risk convergence for stroke (approximately 20 years) versus IHD (> 25 years) observed in the present study.

Compared with current smokers, former smokers experienced a significant reduction in the risk of progression from the health stage to stroke and IHD soon after quitting, whereas T2D and CKD required more than 20 years for similar reductions. Notably, compared with current smokers, T2D risk rose shortly after cessation, consistent with a US-based cohort study reporting increased T2D risk among recent quitters (*HR* = 1.22, 95% *CI*: 1.12–1.32), peaking at 5–7 years after cessation before gradually declining thereafter [19]. This may be partly attributable to weight gain in some individuals shortly after smoking cessation, which can worsen glucose metabolism and insulin resistance [39, 40]. Although previous studies have shown that smoking cessation can substantially reduce the risk of CKD onset and progression [41], evidence on the association between duration since cessation and CKD risk in the general population remains limited. Our data suggest that approximately 23 years are needed for former smokers to match never-smoker CKD risk. This suggests that, although the overall risk of progressing from the health stage to FCRMD begins to decline approximately five years after cessation compared with current smokers, individuals who have recently quit should pay particular attention to the risks of T2D and CKD—especially T2D, which may even show a short-term increase in risk. This highlights the need for additional control of T2D-related risk factors in this group. Building on previous research, our findings further indicate that the beneficial effect of smoking cessation on the risk of progression from the health stage to individual FCRMDs is not influenced by the corresponding genetic risk level, supporting the promotion of smoking cessation across the general population as a strategy for FCRMD risk reduction.

For progression from existing CRM diseases, our findings are consistent with prior studies. Regarding the impact of smoking cessation on the progression from individual CRM diseases to CRMM or death, a Korean cohort study found that, among patients with T2D, quitting smoking significantly reduced the subsequent risk of CVD events and all-cause mortality [23]. Another cohort study of newly diagnosed male diabetes patients in Korea reported that even short-term cessation was associated with a significantly lower risk of future all-cause mortality compared with current smoking [42]. We similarly found mortality differences between former and current smokers as early as five years after cessation. Furthermore, an analysis integrating data from the KNOW-CKD and UK Biobank cohorts showed that in CKD patients, longer cessation duration reduced ASCVD and mortality risk, but even after 20 years, risk remained higher than that in never smokers (*HR* = 1.11, 95% *CI*: 1.06–1.16) [24]. A prospective cohort study based on a US population found that among individuals with CVD, the risk of all-cause mortality decreased significantly with increasing time since cessation; however, even after 20 years, CVD-related mortality risk remained significantly higher than that of never smokers (*HR* = 1.10, 95% *CI*: 1.05–1.16) [43]. Consistent with this, our study showed that for individuals with IHD or stroke as the FCRMD, the risk of direct progression to death after smoking cessation required approximately 25 years to reach the level observed in never smokers, whereas compared with current smokers, a significant reduction in mortality risk was evident after about five years of cessation.

Overall, our results show that for individuals with an existing FCRMD, approximately 23 years of smoking cessation are required for the risks of progression to CRMM and death to decline to a level statistically indistinguishable from that of never smokers. Compared with current smokers, the risk of progression from FCRMD to CRMM decreases significantly only after about 20 years of cessation—considerably longer than the time needed for the risk of progression from FCRMD to death to decline. At the level of specific FCRMDs, patients whose first FCRMD is IHD or CKD should maintain long-term vigilance regarding their risk of developing other CRM diseases or death after cessation. Among them, CKD patients demonstrate an earlier reduction in the risk of progression to CRMM compared with IHD patients. For individuals with diabetes as the FCRMD, the risk of death remains significantly higher than that of never smokers for a longer period than the risk of progression to CRMM. In the stroke population, smoking primarily affects the pathway to death, but the time needed to equalize the risk with that of never smokers is relatively shorter than for other CRM diseases. In addition, compared with light smokers, heavy smokers have markedly higher risks across all CRMM progression pathways, and the benefits of cessation take longer to manifest. Therefore, more urgent smoking cessation interventions should be implemented for heavy smokers to reduce CRMM progression earlier and promote health.

This study has limitations. First, all smoking traits were derived from baseline self-reported questionnaires, and the assessment did not include e-cigarette use, which has become increasingly prevalent in recent years. Therefore, exposure measurement error or misclassification cannot be fully ruled out. Second, this study focused on baseline smoking status and years since cessation to assess subsequent risks across CRMM transition stages, as follow-up information on smoking behavior or relapse was unavailable. Further investigations are warranted to examine the relationship between changes in smoking or quitting behavior and CRMM progression. Third, although we preselected a set of covariates reference to previous literature on CRMM progression and performed sensitivity analysis with a broader set of covariates, residual confounding cannot be excluded. Thus, the association observed in this study should be interpreted with caution when inferring causality. Fourth, the majority of participants in the UK Biobank were of European ancestry, future studies involving diverse ethnic groups are warranted to further validate the extrapolated to other ethnic populations. Finally, participants in the UK Biobank tend to be healthier and exhibit greater health consciousness compared with the broader population; thus, our findings may be subjected to volunteer selection bias, which may also affect generalizability. 

## Conclusions

This study quantified the associations of smoking and cessation behaviors with risk across different stages of CRMM progression. Among all stages of CRMM progression, smoking contributed most substantially to the risk of mortality-related transitions, while the benefits of smoking cessation were also most pronounced in these pathways. Our findings highlight the importance of refining risk assessment across the entire CRMM trajectory by taking past smoking behaviors of former smokers into account, and provide epidemiological evidence to inform the development of targeted prevention and management strategies.

## Supplementary Information

Supplementary Material 1.

## Data Availability

All data used in this study can be downloaded from public UK Biobank Resource ([www.ukbiobank.ac.uk/] (http:/www.ukbiobank.ac.uk)) via application. This study performed using the application number 98273.

## Abbreviations

CRM: Cardio-renal-metabolic
CVD: Cardiovascular disease
T2D: Type 2 diabetes
CKD: Chronic kidney disease
CRMM: CRM multimorbidity
AHA: American Heart Association
FCRMD: First CRM disease
IHD: Ischemic heart disease
ICD-10: International Statistical Classification of Diseases, Tenth Revision
BMI: Body mass index
SBP: Systolic blood pressure
TC: Total cholesterol
HRs: Hazard ratios
CIs: Confidence intervals
GRS: Genetic risk score
STROBE: Strengthening the Reporting of Observational Studies in Epidemiology
IQR: Interquartile range
