# Supplementary material for: Smoking status and cessation duration in relation to the progression of cardio-renal-metabolic multimorbidity: a prospective cohort study from the UK Biobank
Source: Arch Public Health. 2026 Feb 3;84:77. doi: 10.1186/s13690-026-01846-x (PMC13078062; doi:10.1186/s13690-026-01846-x)
Supplement: Supplementary file 1 — Supplementary Material 1. [file 13690_2026_1846_MOESM1_ESM.pdf]

Supplementary Material

Smoking status and cessation duration in relation to the progression of cardio-renal-metabolic multimorbidity: a prospective cohort study from the UK Biobank

Xinhui Liu, Shuo Wu, Heng Zhang*, Fuzhong Xue*

Content

Supplemental Method 1 The definition of covariates 4

Supplemental Method 2 Additional methods 9

Temporal pattern for pathway risk after smoking cessation *9*

Genetic risk scores (GRS) calculation *9*

Table S1 Definitions and descriptions of smoking-related traits in a prospective cohort study from the UK Biobank (baseline assessment 2006–2010). 11

Table S2 Definitions and descriptions of covariates in a prospective cohort study from the UK Biobank (baseline assessment 2006–2010). 12

Table S3 Single nucleotide polymorphisms for the genetic risk score calculation in a prospective cohort study from the UK Biobank (baseline assessment 2006–2010). 15

Table S4 Sensitivity analyses of the associations between smoking behavior and subsequent transitions from first cardio-renal-metabolic disease or cardio-renal-metabolic multimorbidity using multivariable Cox regression models in the UK Biobank prospective cohort (baseline 2006–2010; follow-up through 2022) 17

Table S5 Multiplicative interactions between categorical genetic risk score and smoking status across transition pathways of cardio-renal-metabolic disease progression in a prospective cohort study from the UK Biobank (baseline 2006–2010; follow-up through 2022) 18

Figure S1 Association of smoking status and year since quitting with individual cardio-renal-metabolic disease and cardio-renal-metabolic multimorbidity state compared with current smokers in a prospective cohort study from the UK Biobank (baseline 2006–2010; follow-up through 2022). 20

Figure S2 Risk for individual cardio-renal-metabolic disease and cardio-renal-metabolic multimorbidity state by years since quitting in a prospective cohort study from the UK Biobank (baseline 2006–2010; follow-up through 2022). 21

Figure S3 Association of smoking status and year since quitting with pathways of cardio-renal-metabolic multimorbidity transition pattern comprising four states compared with current smokers in a prospective cohort study from the UK Biobank (baseline 2006–2010; follow-up through 2022). 22

Figure S4 Association of smoking status and year since quitting with pathways in the cardio-renal-metabolic multimorbidity transition pattern comprising four states compared with never smokers adjusting for additional covariates in a prospective cohort study from the UK Biobank (baseline 2006–2010; follow-up through 2022). 23

Figure S5 Association of smoking status and year since quitting with pathways in the cardio-renal-metabolic multimorbidity transition pattern comprising four states compared with never smokers in a prospective cohort study from the UK Biobank (baseline 2006–2010; follow-up through 2022; imputed dataset, N = 469,945) 24

Figure S6 Association of dynamically updated years since smoking cessation with transition pathways of cardio-renal-metabolic multimorbidity in a prospective cohort study from the UK Biobank (baseline 2006–2010; follow-up through 2022). 25

Figure S7 Risk of pathways in the cardio-renal-metabolic multimorbidity transition pattern A by years since quitting in sensitivity analysis restricted to participants with baseline first cardio-renal-metabolic disease or cardio-renal-metabolic multimorbidity population in a prospective cohort study from the UK Biobank (baseline 2006–2010; follow-up through 2022). 27

Figure S8 Association of smoking status and year since quitting with pathways in the cardio-renal-metabolic multimorbidity transition pattern B compared with current smokers in a prospective cohort study from the UK Biobank (baseline 2006–2010; follow-up through 2022). 28

Figure S9 Association of smoking status and year of smoking before quitting with pathways in the cardio-renal-metabolic multimorbidity transition pattern comprising four states compared with never smokers in a prospective cohort study from the UK Biobank (baseline 2006–2010; follow-up through 2022). 29

Figure S10 Association of smoking status and smoking intensity with pathways in the cardio-renal-metabolic multimorbidity transition pattern comprising four states compared with never smokers in a prospective cohort study from the UK Biobank (baseline 2006–2010; follow-up through 2022). 30

Figure S11 Association of smoking status and year since quitting with pathways in the cardio-renal-metabolic multimorbidity transition pattern comprising four states in light smokers (< 20 pack-year) compared with never smokers in a prospective cohort study from the UK Biobank (baseline 2006–2010; follow-up through 2022). 31

Figure S12 Risk for transition of pathways in the cardio-renal-metabolic multimorbidity transition pattern comprising four states by years since quitting in light smokers (< 20 pack-year) in a prospective cohort study from the UK Biobank (baseline 2006–2010; follow-up through 2022). 32

Figure S13 Association of smoking status and year since quitting with pathways in the cardio-renal-metabolic multimorbidity transition pattern comprising four states in heavy smokers (>= 20 pack-year) compared with never smokers in a prospective cohort study from the UK Biobank (baseline 2006–2010; follow-up through 2022). 33

Figure S14 Risk for transition of pathways in the cardio-renal-metabolic multimorbidity transition pattern comprising four states by years since quitting in heavy smokers (>= 20 pack-year) in a prospective cohort study from the UK Biobank (baseline 2006–2010; follow-up through 2022). 34

Figure S15 Association of smoking status and year since quitting with pathways from baseline to each individual first cardio-renal-metabolic disease in the cardio-renal-metabolic multimorbidity transition pattern comprising four states in subgroup with different genetic risk based on corresponding genetic risk score compared with never smokers in a prospective cohort study from the UK Biobank (baseline 2006–2010; follow-up through 2022). 36

Figure S16 Joint effects of smoking status and year since quitting on pathways from baseline to each individual first cardio-renal-metabolic disease in the cardio-renal-metabolic multimorbidity transition pattern comprising four states compared with never smoker with low genetic risk in a prospective cohort study from the UK Biobank (baseline 2006–2010; follow-up through 2022). 37

References 38

Supplemental Method 1 The definition of covariates

The UK Biobank Field ID used for the definitions of all covariates were provided in **Supplemental Table S2**. Specifically, for **physical activity, diet, employment, education, and health sleep score**:

(1) Subjective physical activity[1]

Firstly, total physical activity (TPA) which was a modified version of the International Physical Activity Questionnaire (IPAQ), was assessed through capturing the frequency and duration of walking (Field ID 864 and 874), moderate (Field ID 884 and 894), and vigorous PA (Field ID 904 and 914) performed over the last seven days. Data were analyzed in accordance with the IPAQ scoring protocol. MET levels included walking (3.3), moderate-intensity (4.0), and vigorous-intensity (8.0)[2].

For both the measures, total weekly PA (MET-mins/week) was calculated by multiplying the frequency, duration, and the MET values[2]. Based on the standard scoring criteria of PA, we grouped the sample as low PA (< 600 MET-mins/week), moderate PA (600 to < 3000 MET-mins/week), and high PA (≥ 3000 MET-mins/week); the threshold at 600 MET-mins/week is equivalent to reaching the recommended guidelines (150 minutes per week) for moderate-intensity PA.

(2) Diet

Participant's diet was assessed by their consumption of fruit, vegetable, meat, and fish. To calculate the fruit and vegetable intake, the combined responses for fresh fruit (pieces: one apple, one banana, 10 grapes etc. as one piece, a portion is 1 pieces) (Field ID 1309), dried fruit (pieces: one prune, one dried apricot, 10 raisins as one piece) (Field ID 1319), salad/raw vegetable (heaped tablespoons) (Field ID 1299), and cooked vegetable (heaped tablespoons) (Field ID 1289), were converted into proportions (a portion is 1 piece fresh fruit, 2.5 piece dried fruit, 7 heaped tablespoons salad/raw vegetable, and 3 heaped tablespoons cooked vegetable) and grouped to indicate whether participants ate < 5 or ≥ 5 portions/day, based on the NHS guidelines (http://www.nhs.uk/Livewell/5ADAY/Pages/Portionsizes.aspx), or had missing information.

(3) Employment

Employment status was grouped as working (in paid employment or self-employed (value 1)), unemployed (value 5), retired (value 2), or other (looking after home and/or family (value 3), unable to work because of sickness or disability (value 4), doing unpaid or voluntary work (value 6), full or part time student (value 7). The first entered employment was assumed as their most current employment status[1].

(4) Education

We turn the education qualification into years of education according to International Standard Classification for Education (ISCED) definitions[3]:

Qualification (As reported in UK Biobank)	ISCED	Years of education
College or University degree 1	5	20
NVQ or HND or HNC or equivalent 5	5	19
Other prof. qual. eg: nursing, teaching 6	4	15

A levels/AS levels or equivalent 2	3	13
O levels/GCSEs or equivalent 3	2	10
CSEs or equivalent 4	2	10
None of the above -7	1	7
Prefer not to answer	Excluded	

(5) Healthy sleep score

Based on the study of Fan et al.(2020)[4], five sleep factors (chronotype, duration, insomnia, snoring, and excessive daytime sleepiness) were included to generate a healthy sleep score. Low-risk sleep factors were defined as follows: early chronotype ('morning' or 'morning than evening'); sleep 7–8 h per day; reported never or rarely insomnia symptoms; no self-reported snoring; and no frequent daytime sleepiness ('never/rarely' or 'sometimes'). For each sleep factor, the participant received a score of 1 if he or she was classified as low risk for that factor or 0 if at high risk for that factor. All component scores were summed to obtain a healthy sleep score ranging from 0 to 5, with higher scores indicating a healthier sleep pattern.

For variables about **medication use:** the UK Biobank Field ID and medication name and codes used were listed below:

(1) Statin/cholesterol lowering medication:

Field ID 6153/6177: 1 Cholesterol lowering medication

Field ID 20003:

**Medication Name**	**Code**
Atorvastatin	1141146234
Eptastatin	1140910632
Fluvastatin	1140888594
Pravastatin	1140888648
Rosuvastatin	1141192410
Simvastatin	1140861958
Crestor (rosuvastatin)	1141192414
Lescol (fluvastatin)	1140864592
Lipitor (atorvastatin)	1141146138
Lipostat (pravastatin)	1140861970
Simvador (simvastatin)	1141188146
Zocor (simvastatin)	1140881748
Zocor heart-pro (simvastatin)	1141200040

(2) Anti-hypertension medication

Field ID 6153/6177: 2 Blood pressure medication

Field ID 20003:

**Medication Name**	**Code**	**Medication Name**	**Code**
**Beta blocking agents**			
Acebutolol	1140866724	Nebivolol	1141164276
Atenolol	1140866738	Oxprenolol	1140879830
Bisoprolol	1140879760	Pindolol	1140860292
Carvedilol	1140909368	Propranolol	1140879842

Celiprolol	1140879762	Sotalol	1140879854
Labetalol	1140879824	Angilol (propranolol)	1140866704
Metoprolol	1140879818	Apsolol (propranolol)	1140866764
Nadolol	1140860192	Bedranol (propranolol)	1140851556
Soloc (bisoprolol)	1141182904	Tensomex (metoprolol)	1141182968
Sotacor (sotaloli)	1140860362	Trandate (labetaloli)	1140860250
Tenormin (atenolol)	1140866756	Trasico (oxpernolol)	1140860222
Tensomex (metoprolol)	1141182968	Vasaten (atenolol)	1140866758
Propranolol hydrochloride + bendrofluazide	1140860418	Sotalol hydrochloride + hydrochlorothiazide	1140860332
Beta-cardone (sotalol)	1140860304	Eucardic (carvedilol)	1141168498
Beta-programe (propranolol)	1140866782	Half beta-programe (propranolol)	1140866802
Betaloc (metoprolol)	1140860266	Half propanixla (propranolol)	1141152076
Cardicor (bisoprolol)	1141171152	Half propatard la (propranolol)	1141156754
Cardinol (atenolol)	1140866712	Half-inderal la (propranolol)	1140866800
Celectol (celiprolol)	1140860498	Inderal (propranolol)	1140866804
Corgard (nadolol)	1140860194	Lopresor (metoprolol)	1140860274
Emcor (bisoprolol)	1140860492	Mepranix (metoprolol)	1140860278
Visken (pindolol)	1140860294	Atenolol + chlorthalidone	1141146124
Atenolol + bendrofluazide	1141146126	Atenolol + coamilozide	1141146128
Atenolol + bendroflumethiazide	1141194810	Atenolol + nifedipine	1140860426
Atenolol + chlortalidone	1141180778	Bisoprolol fumarate + hydrochlorothiazide	1140864950
Beta-Adrenoceptor Blocking Drugs	1140866692	Sectral (acebutolol)	1140866726
Monocor (bisoprolol)	1140860434	Slow-trasicor (oxprenolol)	1140860220
Nebilet (nebivolol)	1141164280	Metoprolol tartrate + chlorthalidone	1140860308
Paritane (oxprenolol)	1140851484	Metoprolol tartrate + hydrochlorothiazide	1140860404
Probetala (propranolol)	1140916868	Nadolol + bendrofluazide	1140860312
Propanix (propranolol)	1140866766	Nadolol + bendroflumethiazide	1141194804
Rapranol (propranolol)	1141187048		
**Calcium channel blockers**			

Amlodipine	1140879802	Nicardipine	1140879810
Diltiazem	1140879806	Nifedipine	1140861088
Felodipine	1140888646	Nimodipine	1140872568
Isradipine	1140861190	Verapamil	1140888510
Lacidipine	1140861276	Adalat (nifedipine)	1140861090
Lercanidipine	1141153026	Adulate (nifedipine)	1140881702
Keloc (felodipine)	1141188920	Nimotop (nimodipine)	1140872472
Motens (lacidipine)	1140861282	Parmid (felodipine)	1141201814
Neofel (felodipine)	1141200782	Plendil (felodipine)	1140928212
Nifedipress (nifedipine)	1141157140	Securon (verapamil)	1140866466
Nifedotard (nifedipine)	1141150538	Slofedipine (nifedipine)	1141150500
Nifopress (nifedipine)	1141169730	Slozem (diltiazem)	1140911698
Adipine (nifedipine)	1140923572	Cardene (nicardipine hydrochloride)	1140861176
Adizem-60 (diltiazem)	1140861138	Cardioplen (felodipine)	1141199858
Adizem-xl (diltiazem)	1140926780	Coracten (nifedipine)	1140861120
Angitil (diltiazem)	1140917428	Cordilox (verapamil)	1140866554
Cabren (felodipine)	1141187094	Dilzem (diltiazem)	1140861166
Calchan (nifedipine)	1141173766	Dilcardia (diltiazem)	1141157136
Tensipine (nifedipine)	1140927940	Verapress (verapamil)	1141150926
Tildiem (diltiazem)	1140861128	Vertab (verapamil)	1141169710
Unipine (nifedipine)	1140926188	Zanidip (lercanidipine)	1141153032
Univer (verapamil)	1140881692	Diltiazem hcl + hydrochlorothiazide	1140926778
Vascalpha (felodipine)	1141190160	Felodipine + ramipril	1141165470
Vera-til (verapamil)	1141187774	Half securon (verapamil)	1140866460
Felogen (felodipine)	1141188576	Hypolar (nifedipine)	1141188936
Felotens (felodipine)	1141188152	Istin (amlodipine)	1140861202
Fortipine (nifedipine)	1141145870		
**Agents acting on the renin-angiotensin system**			
Candesartan cilexetil	1141156836	Moexipril	1140923712
Captopril	1140860750	Olmesartan	1141193282
Cilazapril	1140860882	Perindopril	1140888560
Enalapril	1140888552	Quinapril	1140860728
Eprosartan	1141171336	Ramipril	1140860806
Fosinopril	1140888556	Telmisartan	1141166006
Imidapril hydrochloride	1141164148	Trandolapril	1140860904
Indapamide	1140866078	Valsartan	1141145660
Irbesartan	1141152998	Accupro (quinapril)	1140881706
Lisinopril	1140860696	Amias (candesartan)	1141156846
Losartan	1140916356	Aprovel (irbesartan)	1141153006
Capoten (captopril)	1140860758	Olmetec (olmesartan)	1141193346
Carace (lisinopril)	1140864910	Renitec (enalapril)	1140881712

Carace (lisinopril)	1140860706	Staril (fosinopril)	1140860878
Cozaar (losartan)	1141179974	Tanatril (imidapril)	1141164154
Cozaar (losartan)	1140916362	Teveten (eprosartan)	1141171344
Diovan (valsartan)	1141145668	Tritace (ramipril)	1141188408
Gopten (trandolapril)	1140860912	Vascace (cilazapril)	1140860892
Innovace (enalapril)	1140860776	Zestril (lisinopril)	1140860714
Kaplon (captopril)	1141150560	Captopril + hydrochlorothiazide	1140860764
Lopace (ramipril)	1141199940	Cozaar-comp (hydrochlorothiazide + losartan)	1141151018
Micardis (telmisartan)	1141172492	Enalaprilmaleate + hydrochlorothiazide	1140860790
Lisinopril + hydrochlorothiazide	1140864952	Trandolapril+verapamilh ydrochloride	1141153328
Losartan potassium + hydrochlorothiazide	1141151016	Valsartan + hydrochlorothiazide	1141201038
Perindopril + indapamide	1141180592	Angiotensin ii receptor antagonist	1141145658
Tarka (trandolapril + verapamil)	1141153316	Angiotensin ii receptor antagonist + diuretic	1141150898
Telmisartan + hydrochlorothiazide	1141187788		
**Centrally-Acting Antihypertensive Drugs: 1140888578**			

Supplemental Method 2 Additional methods

Temporal pattern for pathway risk after smoking cessation

We explored the temporal pattern and extent of risk decline for each single status after quitting smoking, following the approach used in previous studies[5,6]. Years since quitting were treated as a continuous variable, with durations exceeding 25 years set to 26 (range: 0-26). A Cox model with restricted cubic splines (five knots) was employed to capture potential nonlinear association with the log hazard of each single status. Two analyses were performed: (1) comparing former and current smokers (assigning a value of 0 for years since quitting) to never smokers (for whom years since quitting was set to 50, significantly larger than all former smokers[5,6]); and (2) comparing former smokers to current smokers.

Genetic risk scores (GRS) calculation

Fourth, for each individual FCRMD, we constructed both weighted and unweighted genetic risk scores (GRSs)[4] based on independent single nucleotide polymorphisms (SNPs) that showed significant genome-wide association with IHD[7], stroke[8], T2D[9], and CKD[10]. According to previous study[4] on GRS of coronary heart disease (CHD) and stroke, 74 independent single nucleotide polymorphisms (SNPs) and 10 SNPs that showed significant genome-wide association with CHD and stroke in previous published genome-wide associations studies (GWASs) was used for the calculation for their GRS. For T2D, the genetic association estimates from a recent GWAS of T2D[11] was used, we extracted all genome-wide significant variants associated with T2D at 5×10^{-8} and then filtered this dataset using a clumping distance of 1 Mb and r^2 threshold of 0.001 to generate an independent set of variants (using *ld_clump* function in the *ieugwasr* R package with linkage disequilibrium estimates from the 1,000 Genomes European reference panel). Finally, 224 independent SNPs was used for the T2D GRS calculation. Finally, totally 25 independent SNPs that were associated with CKD at genome-wide significance ($P<5\times10^{-6}$) were clumped by *Su et al. (2025)*[10] at genetic distance of 10,000 kb and a linkage disequilibrium threshold of $r^2 < 0.001$ was used for the GRS calculation. The details for the selected SNPs are given in **Supplementary Table S3.**

Using the selected SNPs, the GRS for IHD, stroke, T2D, and CKD was calculated separately. For each individual cardio-renal-metabolic disease, we used two approaches for the GRS calculation. First, based on previous study[12], the weighted GRS was calculated as the sum of the genome-wide genotypes (recoded as 0, 1, and 2 according to the number of risk alleles), each weighted by corresponding genotype effect size estimates derived from published GWAS summary statistic data (IHD[7], stroke[8], T2D[9], and CKD[10]). Secondly, the unweighted GRS was also calculated as the simple sum of risk alleles across all selected SNPs.

We divided all the participants into high (quintile 5), intermediate (quintile 2–4), or low (quintile 1) genetic risk group based on GRS (weighted and unweighted) for each individual disease. Within each genetic risk category, we used multi-state models to evaluate the associations of smoking status and years since cessation with the risk of progression from the health state to the corresponding FCRMD. We assessed the interaction between the categorical GRS and smoking status on a multiplicative scale using a likelihood ratio test, comparing models with and without a cross-product term. In addition, we estimated the joint effects of GRS and smoking-related exposures, with never smokers in the lowest genetic risk category serving as the reference group.

Table S1 Definitions and descriptions of smoking-related traits in a prospective cohort study from the UK Biobank (baseline assessment 2006–2010).

**Variable**	**Categorizations**	**UK Biobank Field ID**	**Descriptions**
Smoking status	Never, Former, Current	20116 (partially derived from Field 1239 and 1249)	Touchscreen questions, "Do you smoke tobacco now?" and "In the past, how often have you smoked tobacco?"
Years since quitting	Continuous	6194	Touchscreen questionnaire, "How old were you when you last smoked on most days"
Smoking intensity	Light smoker<br>Heavy smoker	20161	The general definition of a pack year is the number of cigarettes smoked per day, divided by twenty, multiplied by the number of years of smoking. The number of years of smoking is calculated by subtracting the age of starting smoking from the age smoking was stopped.
Years of smoking before quitting	Continuous	2867, 2897, 6194	Touchscreen questionnaire, "How old were you when you first started smoking on most days?"<br>"How old were you when you last smoked on most days"

Table S2 Definitions and descriptions of covariates in a prospective cohort study from the UK Biobank (baseline assessment 2006–2010).

**Variable**	**Categorizations**	**UK Biobank Field ID**	**Descriptions**
**Age, years**	Age at baseline	21022	Date attended baseline assessment minus date of birth
**Sex**	Female,<br>Male	31	NHS derived and/or touchscreen questionnaire
**Alcohol intake**	Never,<br>Drinker	1558	Touchscreen questionnaire, "About how often do you drink alcohol?"
**Physical activity level**	Low,<br>Moderate,<br>High	864,<br>874,<br>884,<br>894,<br>904,<br>914	Touchscreen questionnaire,<br>For instance, "In a typical WEEK, on how many days did you walk for at least 10 minutes at a time? (Include walking that you do at work, travelling to and from work, and for sport or leisure)" and "How many minutes did you usually spend walking on a typical DAY?"
**Income levels**	Level 1: Less than £18000,<br>Level 2: £18000 to £30999,<br>Level 3: £31000 to £51999,<br>Level 4: greater than £52000	738	Touchscreen questionnaire, "what is the average total income before tax received by your HOUSEHOLD?
**Employment**	Working,<br>Unemployed,<br>Retired,<br>Other	6142	Touchscreen questionnaire,<br>"Which of the following describes your current situation? (You can select more than one answer)"
**Education**	Years of education < 10 years,<br>≥ 10 years	6138	Touchscreen questionnaire,<br>"Which of the following qualifications do you have?"
**Diet**	< 5 portions/day<br>≥ 5 portions/day	1289,<br>1299,<br>1309,<br>1319	Touchscreen questionnaire,<br>(1)"On average how many heaped tablespoons of COOKED vegetables would you eat per DAY? (Do not include potatoes; put '0' if you do not eat any)"<br>(2) "On average how many heaped tablespoons of SALAD or RAW

			vegetables would you eat per DAY? (Include lettuce, tomato in sandwiches; put '0' if you do not eat any)"<br>(3) "About how many pieces of FRESH fruit would you eat per DAY? (Count one apple, one banana, 10 grapes etc. as one piece; put '0' if you do not eat any)"<br>(4) "About how many pieces of DRIED fruit would you eat per DAY? (Count one prune, one dried apricot, 10 raisins as one piece; put '0' if you do not eat any)"
**Health sleep score**	0,<br>1,<br>2,<br>3,<br>4,<br>5	1160,<br>1180,<br>1200,<br>1210,<br>1220	Touchscreen questionnaire,<br>(1) "Do you consider yourself to be?"<br>(2) "About how many hours sleep do you get in every 24 hours? (Please include naps)"<br>(3) "Do you have trouble falling asleep at night or do you wake up in the middle of the night?"<br>(4) "Does your partner or a close relative or friend complain about your snoring?"<br>(5) "How likely are you to doze off or fall asleep during the daytime when you don't mean to? (e.g., when working, reading or driving)"
**Body mass index, kg/m^2**	Continuous	21001	Physical examination: body mass index
**Systolic blood pressure, mmHg**	Continuous	4080	Physical measures: systolic blood pressure, automated reading
**Total cholesterol, mmol/L**	Continuous	30690	Blood biochemistry: cholesterol
**Antihypertensive medication**	No<br>Yes	6153,<br>6177,<br>20003	Touchscreen questionnaire,<br>"Do you regularly take any of the following medications? (You can

**Statin / other cholesterol lowering medication**	No<br>Yes		select more than one answer)" for 6153 and 6177;<br>20003: Medications health outcomes + self-reported medical conditions

Table S3 Single nucleotide polymorphisms for the genetic risk score calculation in a prospective cohort study from the UK Biobank (baseline assessment 2006–2010).

**Disease**	**Number of SNPs**	**SNPs**	**Weight**
IHD	74	rs11591147, rs56170783, rs7528419, rs11810571, rs6689306, rs67180937, rs16986953, rs585967, rs4299376, rs7568458, rs17678683, rs114123510, rs1250229, rs13003675, rs7623687, rs142695226, rs12493885, rs72627509, rs10857147, rs7678555, rs6841581, rs2306556, rs77335401, rs742115, rs9349379, rs6909752, rs3130683, rs4472337, rs56015508, rs12202017, rs10455872, rs2107595, rs112370447, rs11556924, rs3918226, rs2083636, rs2954029, rs2891168, rs111245230, rs507666, rs1887318, rs1870634, rs2246942, rs11191416, rs10840293, rs3993105, rs2839812, rs964184, rs2229357, rs2681472, rs10774625, rs11830157, rs2244608, rs11057830, rs1924981, rs11617955, rs10139550, rs72743461, rs7164479, rs2083460, rs2071382, rs247616, rs7500448, rs9897596, rs4643373, rs8068952, rs116843064, rs6511720, rs10417115, rs8108632, rs7412, rs1964272, rs28451064, rs180803	Nelson et al. (2017)[7]
Stroke	8	rs2634074, rs2107595, rs10744777, rs2200733, rs7193343, rs12122341, rs11984041, rs12445022	NINDS Stroke Genetics Network (SiGN); International Stroke Genetics Consortium (ISGC). (2016)[8]
T2D	224	rs3768321, rs58432198, rs12140153, rs1127215, rs320369, rs1493694, rs490689, rs3862948, rs9430095, rs340874, rs2820446, rs348330, rs62107261, rs13022337, rs11680058, rs34048824, rs1260326, rs17030845, rs6545714, rs243019, rs2028150, rs11688682, rs16841827, rs7572970, rs13389219, rs2972144, rs11709077, rs1496653, rs4688760, rs891368, rs76263492, rs2292662, rs4368494, rs11708067, rs569255, rs62271373, rs78569745, rs7642311, rs8192675, rs7633675, rs3887925, rs6808574, rs56187241, rs56337234, rs362307, rs10937721, rs7667864, rs13130484, rs79920718, rs1903002, rs6821438, rs10516495, rs7669833, rs28819812, rs745805, rs6885132, rs6884702, rs17261179, rs3811978, rs702634, rs459193, rs2307111, rs7732130, rs1316776, rs145510090, rs115505614, rs329122, rs648795, rs9379084, rs9368222,	Mahajan A, et al. (2022)[9].

		rs3094682, rs2844492, rs601945, rs34298980, rs6905288, rs6937438, rs3798519, rs1665901, rs11759026, rs1573090, rs6557267, rs474513, rs4709746, rs17168486, rs2215383, rs1708302, rs917195, rs878521, rs11496066, rs1562396, rs62492368, rs6459737, rs12542733, rs34990153, rs3021500, rs12680692, rs7819706, rs508419, rs10097617, rs3808415, rs3802177, rs17772814, rs1561927, rs4977213, rs12719778, rs672271, rs10974438, rs62563593, rs2383205, rs10811660, rs1412234, rs12001437, rs17791513, rs2796441, rs55653563, rs505922, rs28533815, rs11257655, rs177045, rs2812539, rs703972, rs10882099, rs144155527, rs34872471, rs2280141, rs4929965, rs231360, rs2237895, rs141521721, rs5215, rs145678014, rs2767036, rs1061810, rs12419690, rs7124681, rs1783541, rs55911137, rs77464186, rs10830963, rs3019208, rs9665898, rs10750397, rs67232546, rs11063029, rs11063069, rs76895963, rs2066827, rs1872992, rs7966976, rs2258238, rs7959830, rs1705263, rs61939481, rs77864822, rs1426371, rs7313918, rs56348580, rs4148856, rs10773051, rs35318451, rs34584161, rs11842871, rs576674, rs9316500, rs9563615, rs1359790, rs7325671, rs17122772, rs17522122, rs8008910, rs2896177, rs3783394, rs34715063, rs2289739, rs2456530, rs144801310, rs11856307, rs7178762, rs4776970, rs13737, rs12910361, rs2351707, rs12910825, rs6600191, rs12325539, rs55872725, rs862320, rs72802358, rs2925979, rs12920022, rs8071043, rs55973554, rs4925109, rs2107133, rs10908278, rs34855406, rs35895680, rs58642235, rs61676547, rs7240767, rs1431841, rs72926932, rs1517037, rs8097210, rs12454712, rs262549, rs4804833, rs10419627, rs739846, rs3786900, rs429358, rs10406431, rs3810291, rs17744783, rs1007090, rs56307709, rs1800961, rs1999536, rs11699802, rs4812034, rs2023681, rs5758223, rs738409, rs36138276	
CKD	25	rs2273368, rs7558579, rs1403450, rs72814059, rs3789126, rs60457579, rs114159997, rs4990988, rs11741692, rs13409, rs9272324, rs118163492, rs10224210, rs11592993, rs1863665, rs10885476, rs7104110, rs192282901, rs7182642, rs11646496, rs77924615, rs62101583, rs7409232, rs6027395, rs4645760	Kurki et al. (2023)[13]

Abbreviations: IHD, ischemic heart disease; T2D, type 2 diabetes; CKD, chronic kidney disease; GRS, genetic risk score; SNPs, single nucleotide polymorphisms.

Table S4 Sensitivity analyses of the associations between smoking behavior and subsequent transitions from first cardio-renal-metabolic disease or cardio-renal-metabolic multimorbidity using multivariable Cox regression models in the UK Biobank prospective cohort (baseline 2006–2010; follow-up through 2022).

		**Never smokers as reference**		**Current smokers as reference**	
	**Case**	***HR* (95% *CI*)**	***P***	***HR* (95% *CI*)**	***P***
**FCRMD to death**					
**Never smokers**	1133	1.00	-	**0.44(0.40,0.48)**	**< 0.001**
**Former smokers**	2081	**1.37(1.27,1.47)**	**< 0.001**	**0.60(0.55,0.65)**	**< 0.001**
≥ 25	509	**1.19(1.07,1.33)**	**0.001**	**0.52(0.46,0.58)**	**< 0.001**
15 to 24	400	**1.38(1.23,1.55)**	**< 0.001**	**0.60(0.53,0.67)**	**< 0.001**
10 to 14	232	**1.51(1.31,1.74)**	**< 0.001**	**0.65(0.56,0.76)**	**< 0.001**
5 to 9	291	**1.57(1.38,1.79)**	**< 0.001**	**0.68(0.59,0.78)**	**< 0.001**
< 5	279	**1.91(1.67,2.18)**	**< 0.001**	**0.83(0.72,0.95)**	**0.006**
**Current smokers**	757	**2.29(2.08,2.52)**	**< 0.001**	1.00	-
**CRMM to death**					
**Never smokers**	297	1.00	-	**0.63(0.52,0.77)**	**< 0.001**
**Former smokers**	600	**1.18(1.02,1.36)**	**0.024**	**0.74(0.62,0.89)**	**0.001**
≥ 25	121	1.00(0.81,1.25)	0.984	**0.63(0.49,0.80)**	**< 0.001**
15 to 24	140	1.22(0.99,1.50)	0.056	**0.77(0.61,0.97)**	**0.024**
10 to 14	66	1.14(0.87,1.50)	0.326	**0.72(0.54,0.96)**	**0.025**
5 to 9	94	**1.37(1.09,1.73)**	**0.008**	0.86(0.67,1.11)	0.254
< 5	59	1.22(0.92,1.61)	0.174	0.76(0.57,1.03)	0.078
**Current smokers**	168	**1.58(1.30,1.92)**	**< 0.001**	1.00	-
**FCRMD to CRMM**					
**Never smokers**	2211	1.00	-	**0.79(0.72,0.85)**	**< 0.001**
**Former smokers**	3058	**1.12(1.06,1.18)**	**< 0.001**	**0.88(0.81,0.95)**	**0.001**
≥ 25	787	**1.10(1.01,1.19)**	**0.031**	**0.86(0.78,0.95)**	**0.003**
15 to 24	559	1.08(0.99,1.19)	0.094	**0.85(0.76,0.95)**	**0.003**
10 to 14	346	**1.27(1.14,1.43)**	**< 0.001**	1.00(0.88,1.13)	0.966
5 to 9	401	**1.15(1.04,1.28)**	**0.009**	0.90(0.80,1.02)	0.093
< 5	372	**1.30(1.16,1.45)**	**< 0.001**	1.02(0.90,1.15)	0.802
**Current smokers**	844	**1.27(1.17,1.38)**	**< 0.001**	1.00	-

All models adjusted for age, sex, employment status, income levels, education, physical activity, diet, healthy sleep score, and alcohol consumption. Abbreviations: *Case*, number of cases; *HR*, hazard ratio; *CI*, confidence interval; *P*, *P* value; CVD, cardiovascular disease; FCRMD, first cardio-renal-metabolic disease; CRMM, cardio-renal-metabolic multimorbidity.

Table S5 Multiplicative interactions between categorical genetic risk score and smoking status across transition pathways of cardio-renal-metabolic disease progression in a prospective cohort study from the UK Biobank (baseline 2006–2010; follow-up through 2022).

Transition	GRS	P for interaction [a]
**Health to T2D**	**Weighted GRS-T2D**	**0.726895**
Health to T2D	Weighted GRS-IHD	0.711728
Health to T2D	Weighted GRS-stroke	0.241845
Health to T2D	Weighted GRS-CKD	0.301227
**Health to T2D**	**Unweighted GRS-T2D**	**0.155337**
Health to T2D	Unweighted GRS-IHD	0.020289
Health to T2D	Unweighted GRS-stroke	0.212400
Health to T2D	Unweighted GRS-CKD	0.746647
Health to IHD	Weighted GRS-T2D	0.046275
**Health to IHD**	**Weighted GRS-IHD**	**0.123149**
Health to IHD	Weighted GRS-stroke	0.755165
Health to IHD	Weighted GRS-CKD	0.375221
Health to IHD	Unweighted GRS-T2D	0.023775
**Health to IHD**	**Unweighted GRS-IHD**	**0.326028**
Health to IHD	Unweighted GRS-stroke	0.400380
Health to IHD	Unweighted GRS-CKD	0.086876
Health to Stroke	Weighted GRS-T2D	0.859244
Health to Stroke	Weighted GRS-IHD	0.225903
**Health to Stroke**	**Weighted GRS-stroke**	**0.281407**
Health to Stroke	Weighted GRS-CKD	0.160212
Health to Stroke	Unweighted GRS-T2D	0.592897
Health to Stroke	Unweighted GRS-IHD	0.244660
**Health to Stroke**	**Unweighted GRS-stroke**	**0.128571**
Health to Stroke	Unweighted GRS-CKD	0.456508
Health to CKD	Weighted GRS-T2D	0.069974
Health to CKD	Weighted GRS-IHD	0.508510
Health to CKD	Weighted GRS-stroke	0.370017
**Health to CKD**	**Weighted GRS-CKD**	**0.523370**
Health to CKD	Unweighted GRS-T2D	0.549413
Health to CKD	Unweighted GRS-IHD	0.447715
Health to CKD	Unweighted GRS-stroke	0.312594
**Health to CKD**	**Unweighted GRS-CKD**	**0.862386**
**Health to T2D**	**Weighted GRS-T2D**	**0.726895**
Health to T2D	Weighted GRS-IHD	0.711728
Health to T2D	Weighted GRS-stroke	0.241845
Health to T2D	Weighted GRS-CKD	0.301227
**Health to T2D**	**Unweighted GRS-T2D**	**0.155337**

Health to T2D	Unweighted GRS-IHD	0.020289
Health to T2D	Unweighted GRS-stroke	0.212400
Health to T2D	Unweighted GRS-CKD	0.746647
Health to IHD	Weighted GRS-T2D	0.046275
**Health to IHD**	**Weighted GRS-IHD**	**0.123149**
Health to IHD	Weighted GRS-stroke	0.755165
Health to IHD	Weighted GRS-CKD	0.375221
Health to IHD	Unweighted GRS-T2D	0.023775
**Health to IHD**	**Unweighted GRS-IHD**	**0.326028**
Health to IHD	Unweighted GRS-stroke	0.400380
Health to IHD	Unweighted GRS-CKD	0.086876
Health to Stroke	Weighted GRS-T2D	0.859244
Health to Stroke	Weighted GRS-IHD	0.225903
**Health to Stroke**	**Weighted GRS-stroke**	**0.281407**
Health to Stroke	Weighted GRS-CKD	0.160212
Health to Stroke	Unweighted GRS-T2D	0.592897
Health to Stroke	Unweighted GRS-IHD	0.244660
**Health to Stroke**	**Unweighted GRS-stroke**	**0.128571**
Health to Stroke	Unweighted GRS-CKD	0.456508
Health to CKD	Weighted GRS-T2D	0.069974
Health to CKD	Weighted GRS-IHD	0.508510
Health to CKD	Weighted GRS-stroke	0.370017
**Health to CKD**	**Weighted GRS-CKD**	**0.523370**
Health to CKD	Unweighted GRS-T2D	0.549413
Health to CKD	Unweighted GRS-IHD	0.447715
Health to CKD	Unweighted GRS-stroke	0.312594
**Health to CKD**	**Unweighted GRS-CKD**	**0.862386**

Multi-state models adjusted for age, sex, alcohol consumption, physical activity, diet, employment status, healthy sleep score, income levels, and education, smoking status.

[a] *P* value for interaction between GRS (low, medium and high) and smoking status (never, former, and current) on the multiplicative scale was evaluated by likelihood ratio test comparing models with and without a cross-product term in the Cox regression models.

Abbreviations: IHD, ischemic heart disease; T2D, type 2 diabetes; CKD, chronic kidney disease; GRS, genetic risk score.

Outcome	Exposure	N	Case	HR (95% CI)
T2D	Never Smokers	37581	2973	0.75 (0.71-0.78)
	Former Smokers	196131	9770	0.93 (0.89-0.97)
	Former Smokers- >= 25 years since quitting	29471	2268	0.82 (0.78-0.87)
	Former Smokers-15-24 years since quitting	20357	1745	1.07 (1.01-1.14)
	Former Smokers-10-14 years since quitting	9136	771	1.10 (1.02-1.20)
	Former Smokers-5-9 years since quitting	10492	990	1.25 (1.17-1.35)
	Former Smokers-< 5 years since quitting	11211	975	1.13 (1.05-1.21)
IHD	Never Smokers	37581	3923	0.64 (0.62-0.67)
	Former Smokers	196131	12367	0.76 (0.74-0.79)
	Former Smokers- >= 25 years since quitting	29471	3207	0.72 (0.68-0.75)
	Former Smokers-15-24 years since quitting	20357	2078	0.84 (0.80-0.89)
	Former Smokers-10-14 years since quitting	9136	896	0.88 (0.81-0.94)
	Former Smokers-5-9 years since quitting	10492	1030	0.90 (0.84-0.97)
	Former Smokers-< 5 years since quitting	11211	1068	0.91 (0.85-0.98)
Stroke	Never Smokers	37581	1207	0.59 (0.55-0.63)
	Former Smokers	196131	3707	0.65 (0.61-0.70)
	Former Smokers- >= 25 years since quitting	29471	871	0.58 (0.53-0.63)
	Former Smokers-15-24 years since quitting	20357	566	0.70 (0.63-0.77)
	Former Smokers-10-14 years since quitting	9136	241	0.73 (0.64-0.84)
	Former Smokers-5-9 years since quitting	10492	255	0.70 (0.61-0.80)
	Former Smokers-< 5 years since quitting	11211	295	0.80 (0.71-0.91)
CKD	Never Smokers	37581	1510	0.75 (0.71-0.80)
	Former Smokers	196131	6094	0.87 (0.83-0.93)
	Former Smokers- >= 25 years since quitting	29471	1586	0.82 (0.76-0.88)
	Former Smokers-15-24 years since quitting	20357	978	0.95 (0.88-1.03)
	Former Smokers-10-14 years since quitting	9136	410	0.99 (0.88-1.10)
	Former Smokers-5-9 years since quitting	10492	456	0.99 (0.89-1.10)
	Former Smokers-< 5 years since quitting	11211	517	1.11 (1.01-1.23)
CVD	Never Smokers	37581	4857	0.63 (0.61-0.65)
	Former Smokers	196131	15355	0.74 (0.71-0.76)
	Former Smokers- >= 25 years since quitting	29471	3895	0.69 (0.66-0.72)
	Former Smokers-15-24 years since quitting	20357	2492	0.80 (0.77-0.84)
	Former Smokers-10-14 years since quitting	9136	1079	0.84 (0.79-0.90)
	Former Smokers-5-9 years since quitting	10492	1225	0.86 (0.81-0.91)
	Former Smokers-< 5 years since quitting	11211	1297	0.89 (0.84-0.94)
FCRMD	Never Smokers	37581	7578	0.70 (0.68-0.72)
	Former Smokers	196131	26364	0.82 (0.80-0.84)
	Former Smokers- >= 25 years since quitting	29471	6340	0.77 (0.74-0.79)
	Former Smokers-15-24 years since quitting	20357	4174	0.91 (0.87-0.94)
	Former Smokers-10-14 years since quitting	9136	1795	0.93 (0.88-0.98)
	Former Smokers-5-9 years since quitting	10492	2159	1.00 (0.96-1.05)
	Former Smokers-< 5 years since quitting	11211	2254	1.00 (0.96-1.05)
CVD&T2D	Never Smokers	37581	927	0.60 (0.55-0.65)
	Former Smokers	196131	2353	0.79 (0.73-0.86)
	Former Smokers- >= 25 years since quitting	29471	654	0.67 (0.60-0.74)
	Former Smokers-15-24 years since quitting	20357	530	0.97 (0.87-1.08)
	Former Smokers-10-14 years since quitting	9136	244	1.07 (0.93-1.23)
	Former Smokers-5-9 years since quitting	10492	271	1.06 (0.92-1.21)
	Former Smokers-< 5 years since quitting	11211	261	0.96 (0.83-1.10)
T2D&CKD	Never Smokers	37581	432	0.61 (0.55-0.68)
	Former Smokers	196131	1258	0.82 (0.74-0.92)
	Former Smokers- >= 25 years since quitting	29471	364	0.70 (0.61-0.81)
	Former Smokers-15-24 years since quitting	20357	268	0.96 (0.82-1.12)
	Former Smokers-10-14 years since quitting	9136	120	1.05 (0.86-1.29)
	Former Smokers-5-9 years since quitting	10492	128	1.01 (0.83-1.23)
	Former Smokers-< 5 years since quitting	11211	147	1.11 (0.92-1.34)
CVD&CKD	Never Smokers	37581	602	0.58 (0.53-0.64)
	Former Smokers	196131	1770	0.76 (0.69-0.83)
	Former Smokers- >= 25 years since quitting	29471	548	0.66 (0.59-0.74)
	Former Smokers-15-24 years since quitting	20357	368	0.87 (0.76-0.99)
	Former Smokers-10-14 years since quitting	9136	158	0.93 (0.78-1.10)
	Former Smokers-5-9 years since quitting	10492	159	0.85 (0.72-1.02)
	Former Smokers-< 5 years since quitting	11211	204	1.10 (0.94-1.29)
CVD&CKD&T2D	Never Smokers	37581	199	0.57 (0.48-0.67)
	Former Smokers	196131	526	0.77 (0.65-0.91)
	Former Smokers- >= 25 years since quitting	29471	157	0.62 (0.50-0.77)
	Former Smokers-15-24 years since quitting	20357	125	0.94 (0.75-1.18)
	Former Smokers-10-14 years since quitting	9136	57	1.06 (0.79-1.42)
	Former Smokers-5-9 years since quitting	10492	46	0.77 (0.56-1.07)
	Former Smokers-< 5 years since quitting	11211	77	1.26 (0.97-1.63)
CRMM	Never Smokers	37581	1723	0.60 (0.56-0.63)
	Former Smokers	196131	4781	0.77 (0.73-0.82)
	Former Smokers- >= 25 years since quitting	29471	1352	0.66 (0.61-0.71)
	Former Smokers-15-24 years since quitting	20357	1004	0.92 (0.85-0.99)
	Former Smokers-10-14 years since quitting	9136	444	0.99 (0.89-1.10)
	Former Smokers-5-9 years since quitting	10492	493	0.99 (0.89-1.09)
	Former Smokers-< 5 years since quitting	11211	492	0.95 (0.86-1.05)
CVD Death	Never Smokers	37581	1047	0.41 (0.38-0.44)
	Former Smokers	196131	1875	0.48 (0.44-0.52)
	Former Smokers- >= 25 years since quitting	29471	468	0.37 (0.33-0.41)
	Former Smokers-15-24 years since quitting	20357	348	0.52 (0.46-0.58)
	Former Smokers-10-14 years since quitting	9136	161	0.58 (0.49-0.68)
	Former Smokers-5-9 years since quitting	10492	197	0.64 (0.55-0.74)
	Former Smokers-< 5 years since quitting	11211	239	0.77 (0.66-0.88)
Non-CVD Death	Never Smokers	37581	4044	0.42 (0.40-0.44)
	Former Smokers	196131	8686	0.55 (0.53-0.57)
	Former Smokers- >= 25 years since quitting	29471	2300	0.46 (0.44-0.48)
	Former Smokers-15-24 years since quitting	20357	1543	0.57 (0.54-0.61)
	Former Smokers-10-14 years since quitting	9136	764	0.70 (0.65-0.76)
	Former Smokers-5-9 years since quitting	10492	926	0.77 (0.71-0.82)
	Former Smokers-< 5 years since quitting	11211	1076	0.87 (0.82-0.93)
All Death	Never Smokers	37581	5060	0.40 (0.39-0.42)
	Former Smokers	196131	10483	0.52 (0.50-0.54)
	Former Smokers- >= 25 years since quitting	29471	2745	0.43 (0.41-0.45)
	Former Smokers-15-24 years since quitting	20357	1881	0.55 (0.52-0.58)
	Former Smokers-10-14 years since quitting	9136	916	0.66 (0.62-0.71)
	Former Smokers-5-9 years since quitting	10492	1116	0.73 (0.69-0.78)
	Former Smokers-< 5 years since quitting	11211	1307	0.85 (0.80-0.90)

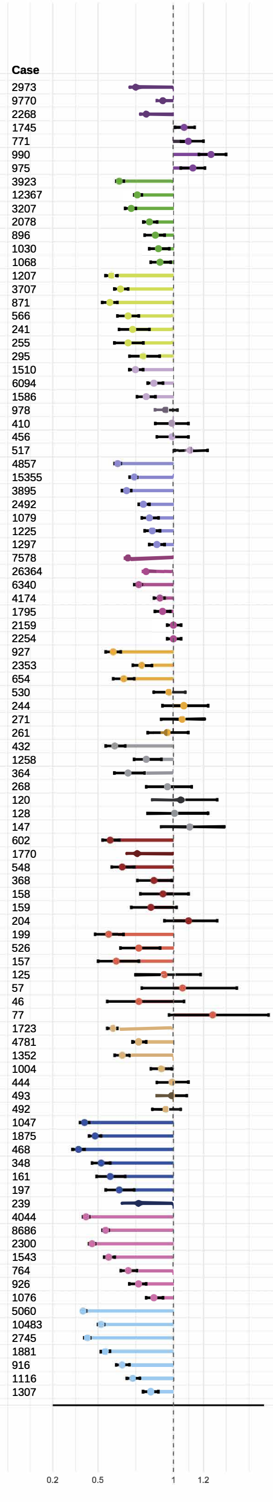

Figure S1 Association of smoking status and year since quitting with individual cardio-renal-metabolic disease and cardio-renal-metabolic multimorbidity state compared with current smokers in a prospective cohort study from the UK Biobank (baseline 2006–2010; follow-up through 2022).

Cox regression models for the association between smoking behavior and each CRMM state;

All models were adjusted for age, sex, alcohol consumption, physical activity, diet, employment status, healthy sleep score, income levels, and education. CVD was defined as the combined outcome of IHD and stroke.

Abbreviations: *N*, total sample size; Case, number of individuals experience the transition; *HR*, hazard ratio; *CI*, confidence interval; *P*, *P* value; Ref. reference group; IHD, ischemic heart disease; T2D, type 2 diabetes; CKD, chronic kidney disease; CVD, cardiovascular disease; FCRMD, first cardio-renal-metabolic disease; CRMM, cardio-renal-metabolic multimorbidity.

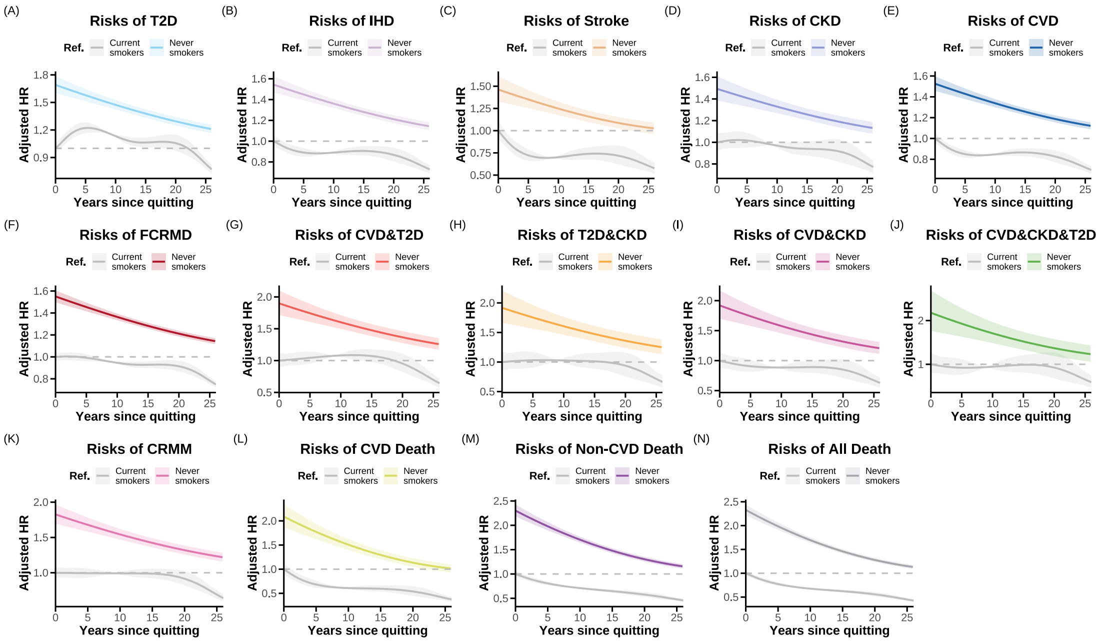

Figure S2 Risk for individual cardio-renal-metabolic disease and cardio-renal-metabolic multimorbidity state by years since quitting in a prospective cohort study from the UK Biobank (baseline 2006–2010; follow-up through 2022).

Restricted cubic splines with five knots were employed to capture potential nonlinear associations with the log hazard of the risk for transition of each pathway. All models were adjusted for age, sex, alcohol consumption, physical activity, diet, employment status, healthy sleep score, income levels, and education. CVD was defined as the combined outcome of IHD and stroke.

Abbreviations: *N*, total sample size; Case, number of individuals experience the transition; *HR*, hazard ratio; *CI*, confidence interval; *P*, *P* value; Ref. reference group; IHD, ischemic heart disease; T2D, type 2 diabetes; CKD, chronic kidney disease; CVD, cardiovascular disease; FCRMD, first cardio-renal-metabolic disease; CRMM, cardio-renal-metabolic multimorbidity.

Exposure	N	Case	HR (95% CI)	P value
Never Smokers	37581	7578	0.70 (0.68-0.72)	<0.001
Former Smokers	196131	26364	0.82 (0.80-0.84)	<0.001
Former Smokers->= 25 years since quitting	29471	6340	0.77 (0.74-0.79)	<0.001
Former Smokers-15-24 years since quitting	20357	4174	0.91 (0.87-0.94)	<0.001
Former Smokers-10-14 years since quitting	9136	1795	0.93 (0.88-0.98)	0.007
Former Smokers-5-9 years since quitting	10492	2159	1.00 (0.96-1.05)	0.867
Former Smokers-< 5 years since quitting	11211	2254	1.00 (0.96-1.05)	0.931
Never Smokers	37581	3217	0.40 (0.39-0.42)	<0.001
Former Smokers	196131	7030	0.50 (0.48-0.52)	<0.001
Former Smokers->= 25 years since quitting	29471	1669	0.42 (0.40-0.45)	<0.001
Former Smokers-15-24 years since quitting	20357	1061	0.51 (0.47-0.54)	<0.001
Former Smokers-10-14 years since quitting	9136	551	0.64 (0.59-0.70)	<0.001
Former Smokers-5-9 years since quitting	10492	680	0.72 (0.66-0.78)	<0.001
Former Smokers-< 5 years since quitting	11211	773	0.80 (0.74-0.87)	<0.001
Never Smokers	7578	1723	0.78 (0.73-0.82)	<0.001
Former Smokers	26364	4781	0.86 (0.81-0.91)	<0.001
Former Smokers->= 25 years since quitting	6340	1352	0.75 (0.70-0.81)	<0.001
Former Smokers-15-24 years since quitting	4174	1004	0.92 (0.85-1.00)	0.038
Former Smokers-10-14 years since quitting	1795	444	1.02 (0.92-1.13)	0.751
Former Smokers-5-9 years since quitting	2159	493	0.94 (0.85-1.04)	0.237
Former Smokers-< 5 years since quitting	2254	492	0.93 (0.84-1.03)	0.146
Never Smokers	7578	1325	0.48 (0.45-0.51)	<0.001
Former Smokers	26364	2491	0.57 (0.53-0.61)	<0.001
Former Smokers->= 25 years since quitting	6340	749	0.46 (0.42-0.50)	<0.001
Former Smokers-15-24 years since quitting	4174	520	0.56 (0.50-0.62)	<0.001
Former Smokers-10-14 years since quitting	1795	241	0.66 (0.58-0.76)	<0.001
Former Smokers-5-9 years since quitting	2159	311	0.73 (0.64-0.82)	<0.001
Former Smokers-< 5 years since quitting	2254	375	0.89 (0.79-1.00)	0.044
Never Smokers	1723	518	0.57 (0.51-0.64)	<0.001
Former Smokers	4781	962	0.67 (0.60-0.74)	<0.001
Former Smokers->= 25 years since quitting	1352	327	0.55 (0.48-0.64)	<0.001
Former Smokers-15-24 years since quitting	1004	300	0.76 (0.66-0.88)	<0.001
Former Smokers-10-14 years since quitting	444	124	0.69 (0.57-0.84)	<0.001
Former Smokers-5-9 years since quitting	493	125	0.71 (0.58-0.86)	<0.001
Former Smokers-< 5 years since quitting	492	159	0.89 (0.74-1.06)	0.196

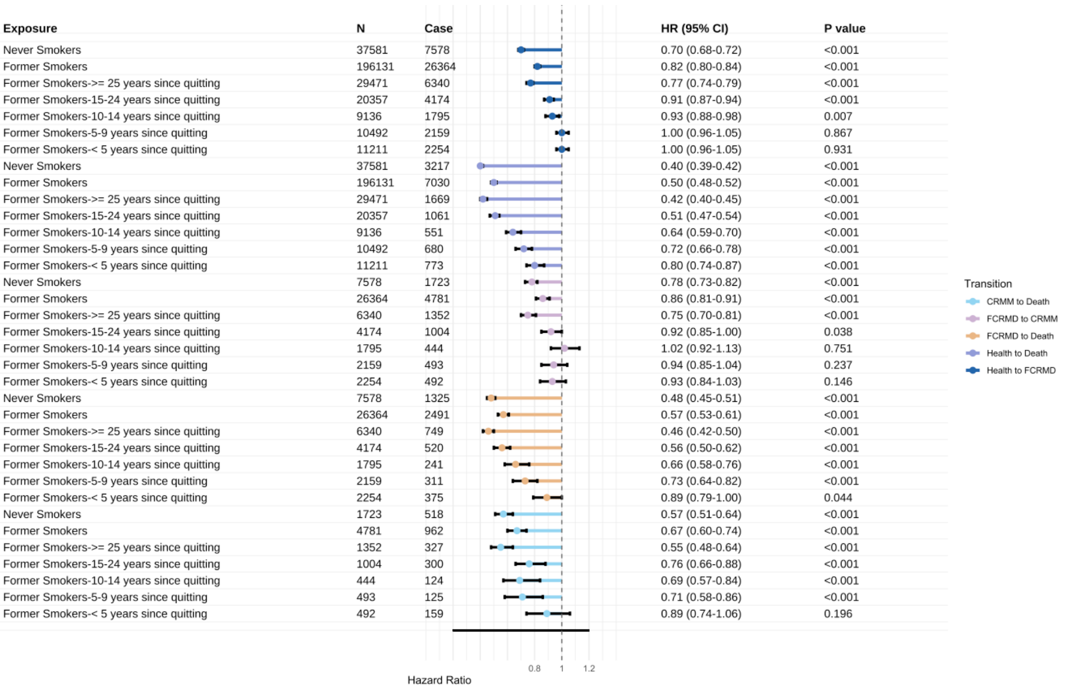

Figure S3 Association of smoking status and year since quitting with pathways of cardio-renal-metabolic multimorbidity transition pattern comprising four states compared with current smokers in a prospective cohort study from the UK Biobank (baseline 2006–2010; follow-up through 2022).

Multi-state models adjusted for age, sex, alcohol consumption, physical activity, diet, employment status, healthy sleep score, income levels, and education.

Abbreviations: *N*, total sample size; Case, number of individuals experience the transition; *HR*, hazard ratio; *CI*, confidence interval; *P*, *P* value; FCRMD, first cardio-renal-metabolic disease; CRMM, cardio-renal-metabolic multimorbidity.

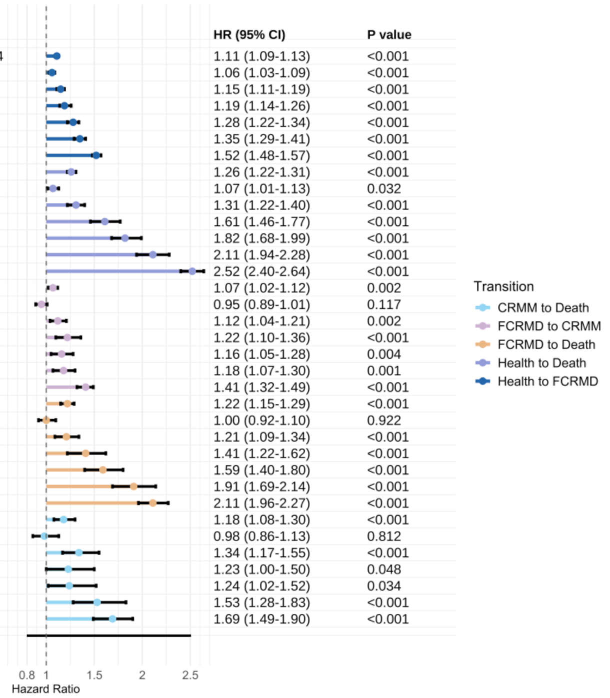

Exposure	N	Case	HR (95% CI)	P value
Former Smokers	122359	22844	1.11 (1.09-1.13)	<0.001
Former Smokers- >= 25 years since quitting	29471	6340	1.06 (1.03-1.09)	<0.001
Former Smokers-15-24 years since quitting	20357	4174	1.15 (1.11-1.19)	<0.001
Former Smokers-10-14 years since quitting	9136	1795	1.19 (1.14-1.26)	<0.001
Former Smokers-5-9 years since quitting	10492	2159	1.28 (1.22-1.34)	<0.001
Former Smokers-< 5 years since quitting	11211	2254	1.35 (1.29-1.41)	<0.001
Current Smokers	37581	7578	1.52 (1.48-1.57)	<0.001
Former Smokers	122359	6437	1.26 (1.22-1.31)	<0.001
Former Smokers- >= 25 years since quitting	29471	1669	1.07 (1.01-1.13)	0.032
Former Smokers-15-24 years since quitting	20357	1061	1.31 (1.22-1.40)	<0.001
Former Smokers-10-14 years since quitting	9136	551	1.61 (1.46-1.77)	<0.001
Former Smokers-5-9 years since quitting	10492	680	1.82 (1.68-1.99)	<0.001
Former Smokers-< 5 years since quitting	11211	773	2.11 (1.94-2.28)	<0.001
Current Smokers	37581	3217	2.52 (2.40-2.64)	<0.001
Former Smokers	22844	5004	1.07 (1.02-1.12)	0.002
Former Smokers- >= 25 years since quitting	6340	1352	0.95 (0.89-1.01)	0.117
Former Smokers-15-24 years since quitting	4174	1004	1.12 (1.04-1.21)	0.002
Former Smokers-10-14 years since quitting	1795	444	1.22 (1.10-1.36)	<0.001
Former Smokers-5-9 years since quitting	2159	493	1.16 (1.05-1.28)	0.004
Former Smokers-< 5 years since quitting	2254	492	1.18 (1.07-1.30)	0.001
Current Smokers	7578	1723	1.41 (1.32-1.49)	<0.001
Former Smokers	22844	2848	1.22 (1.15-1.29)	<0.001
Former Smokers- >= 25 years since quitting	6340	749	1.00 (0.92-1.10)	0.922
Former Smokers-15-24 years since quitting	4174	520	1.21 (1.09-1.34)	<0.001
Former Smokers-10-14 years since quitting	1795	241	1.41 (1.22-1.62)	<0.001
Former Smokers-5-9 years since quitting	2159	311	1.59 (1.40-1.80)	<0.001
Former Smokers-< 5 years since quitting	2254	375	1.91 (1.69-2.14)	<0.001
Current Smokers	7578	1325	2.11 (1.96-2.27)	<0.001
Former Smokers	5004	1316	1.18 (1.08-1.30)	<0.001
Former Smokers- >= 25 years since quitting	1352	327	0.98 (0.86-1.13)	0.812
Former Smokers-15-24 years since quitting	1004	300	1.34 (1.17-1.55)	<0.001
Former Smokers-10-14 years since quitting	444	124	1.23 (1.00-1.50)	0.048
Former Smokers-5-9 years since quitting	493	125	1.24 (1.02-1.52)	0.034
Former Smokers-< 5 years since quitting	492	159	1.53 (1.28-1.83)	<0.001
Current Smokers	1723	518	1.69 (1.49-1.90)	<0.001

Figure S4 Association of smoking status and year since quitting with pathways in the cardio-renal-metabolic multimorbidity transition pattern comprising four states compared with never smokers adjusting for additional covariates in a prospective cohort study from the UK Biobank (baseline 2006–2010; follow-up through 2022).

Multi-state models adjusted for age, sex, alcohol consumption, physical activity, diet, employment status, healthy sleep score, income levels, and education, body mass index, systolic blood pressure, total cholesterol, the use of antihypertensive and statin/other cholesterol-lowering medication.

Abbreviations: *N*, total sample size; Case, number of individuals experience the transition; *HR*, hazard ratio; *CI*, confidence interval; *P*, *P* value; FCRMD, first cardio-renal-metabolic disease; CRMM, cardio-renal-metabolic multimorbidity.

Exposure	N	Case	HR (95% CI)	P value
Former Smokers	158980	30862	1.18 (1.16-1.20)	<0.001
>= 25 years since quitting	38379	8424	1.10 (1.08-1.13)	<0.001
15-24 years since quitting	26042	5495	1.31 (1.27-1.34)	<0.001
10-14 years since quitting	11878	2470	1.37 (1.32-1.43)	<0.001
5-9 years since quitting	13534	2923	1.47 (1.42-1.53)	<0.001
< 5 years since quitting	14569	3103	1.48 (1.43-1.54)	<0.001
Current Smokers	49287	10375	1.45 (1.42-1.49)	<0.001
Former Smokers	158980	8801	1.27 (1.23-1.31)	<0.001
>= 25 years since quitting	38379	2285	1.08 (1.03-1.13)	0.001
15-24 years since quitting	26042	1440	1.31 (1.24-1.38)	<0.001
10-14 years since quitting	11878	732	1.59 (1.48-1.72)	<0.001
5-9 years since quitting	13534	887	1.77 (1.65-1.89)	<0.001
< 5 years since quitting	14569	1068	2.07 (1.95-2.21)	<0.001
Current Smokers	49287	4373	2.52 (2.43-2.62)	<0.001
Former Smokers	30862	6876	1.11 (1.07-1.15)	<0.001
>= 25 years since quitting	8424	1819	0.98 (0.93-1.04)	0.555
15-24 years since quitting	5495	1310	1.17 (1.10-1.24)	<0.001
10-14 years since quitting	2470	621	1.30 (1.20-1.42)	<0.001
5-9 years since quitting	2923	698	1.25 (1.15-1.35)	<0.001
< 5 years since quitting	3103	696	1.19 (1.10-1.29)	<0.001
Current Smokers	10375	2407	1.31 (1.25-1.37)	<0.001
Former Smokers	30862	3956	1.20 (1.15-1.26)	<0.001
>= 25 years since quitting	8424	1032	1.00 (0.93-1.07)	0.997
15-24 years since quitting	5495	714	1.21 (1.11-1.31)	<0.001
10-14 years since quitting	2470	332	1.35 (1.20-1.51)	<0.001
5-9 years since quitting	2923	427	1.51 (1.36-1.67)	<0.001
< 5 years since quitting	3103	510	1.78 (1.62-1.95)	<0.001
Current Smokers	10375	1840	2.09 (1.97-2.21)	<0.001
Former Smokers	6876	1842	1.18 (1.10-1.27)	<0.001
>= 25 years since quitting	1819	456	1.02 (0.91-1.13)	0.770
15-24 years since quitting	1310	385	1.31 (1.16-1.46)	<0.001
10-14 years since quitting	621	174	1.20 (1.02-1.40)	0.025
5-9 years since quitting	698	185	1.25 (1.07-1.46)	0.004
< 5 years since quitting	696	224	1.60 (1.38-1.84)	<0.001
Current Smokers	2407	758	1.80 (1.64-1.97)	<0.001

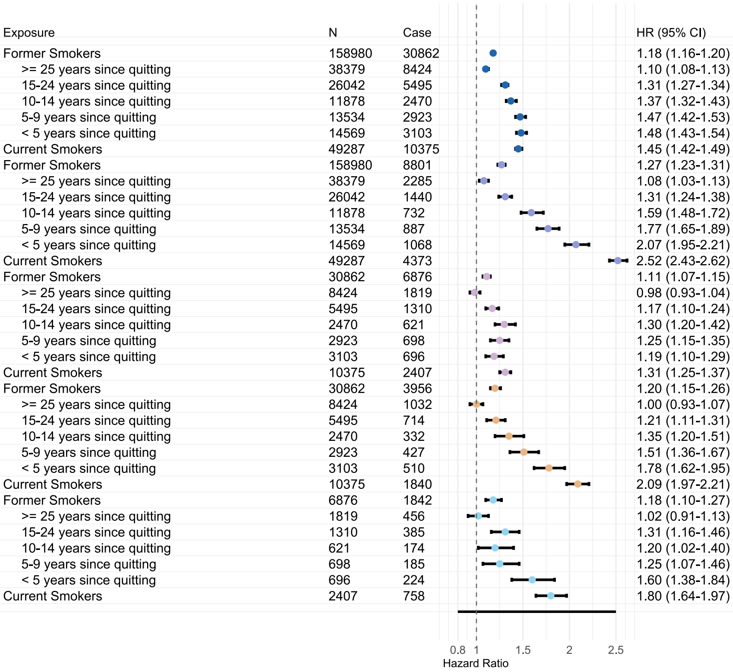

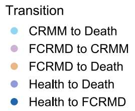

Figure S5 Association of smoking status and year since quitting with pathways in the cardio-renal-metabolic multimorbidity transition pattern comprising four states compared with never smokers in a prospective cohort study from the UK Biobank (baseline 2006–2010; follow-up through 2022; imputed dataset, N = 469,945).

Multi-state models adjusted for age, sex, alcohol consumption, physical activity, diet, employment status, healthy sleep score, income levels, and education, body mass index, systolic blood pressure, total cholesterol, the use of antihypertensive and statin/other cholesterol-lowering medication.

Abbreviations: *N*, total sample size; Case, number of individuals experience the transition; *HR*, hazard ratio; *CI*, confidence interval; *P*, *P* value; FCRMD, first cardio-renal-metabolic disease; CRMM, cardio-renal-metabolic multimorbidity.

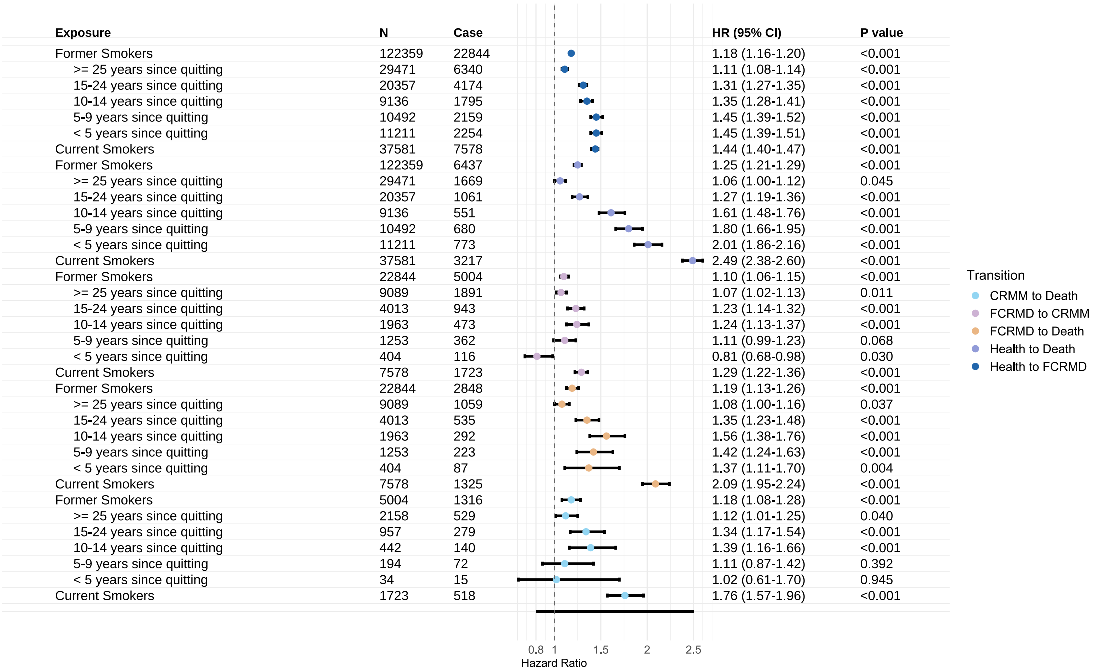

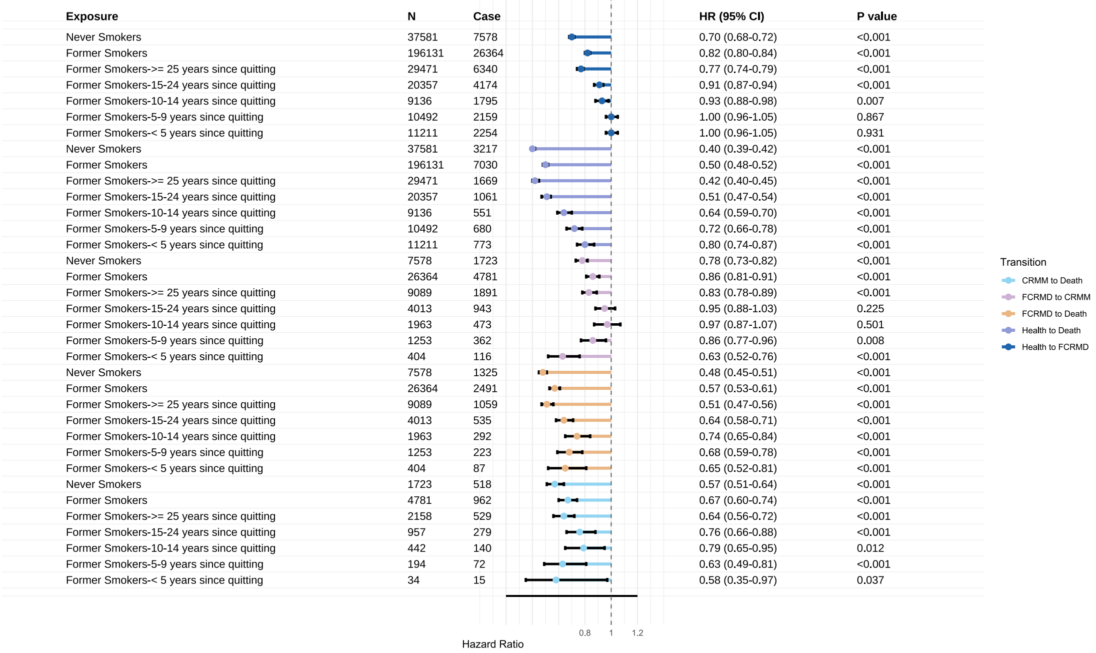

Figure S6 Association of dynamically updated years since smoking cessation with transition pathways of cardio-renal-metabolic multimorbidity in a prospective cohort study from the UK Biobank (baseline 2006–2010; follow-up through 2022).

Multi-state model with time-updated exposure was used to assess the associations of dynamically updated years since smoking cessation with the temporal disease progression from baseline (free of CRMD) to FCRMD, CRMM, and ultimately to death.

For each transition, years since cessation was recalculated dynamically according to the start time of the corresponding transition state to reflect time-dependent exposure duration. This analysis was conducted as a sensitivity analysis to assess the robustness of associations under a time-dependent exposure framework.

All models were adjusted for age, sex, alcohol consumption, physical activity, diet, employment status, healthy sleep score, income levels, and education.

Abbreviations: *N*, total sample size; Case, number of individuals experiencing the transition; *HR*, hazard ratio; *CI*, confidence interval; *P*, *P* value; FCRMD, first cardio-renal-metabolic disease; CRMM, cardio-renal-metabolic multimorbidity. Transition pattern A, transition pathways based on four states in CRMM progression (see Figure 1B).

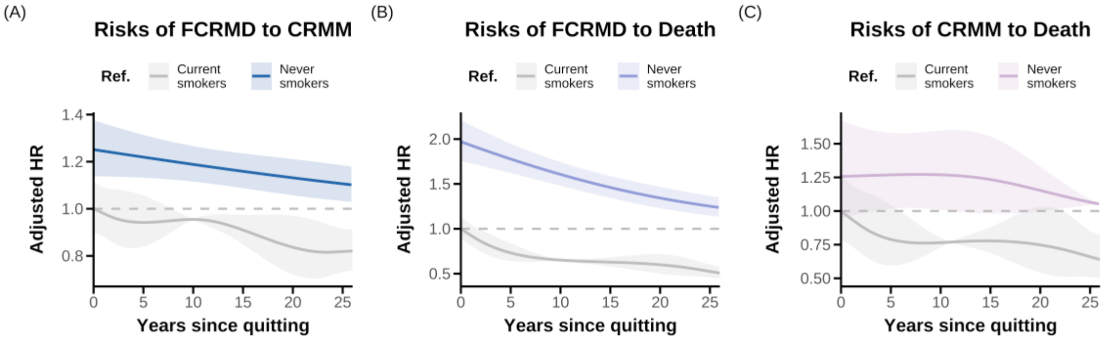

Figure S7 Risk of pathways in the cardio-renal-metabolic multimorbidity transition pattern A by years since quitting in sensitivity analysis restricted to participants with baseline first cardio-renal-metabolic disease or cardio-renal-metabolic multimorbidity population in a prospective cohort study from the UK Biobank (baseline 2006–2010; follow-up through 2022).

Restricted cubic splines with five knots were employed to capture potential nonlinear associations with the log hazard of the risk for each transition pathway. Years since quitting were treated as a continuous variable, with durations exceeding 25 years set to 26 (range: 0-26). Two analyses were performed: (1) comparing former and current smokers (assigning a value of 0 for years since quitting) to never smokers (for whom years since quitting was set to 50, significantly larger than all former smokers following the approach used in previous studies), shown as colored lines in the figure; and (2) comparing former smokers to current smokers, shown as gray lines in the figure.
All models were adjusted for age, sex, alcohol consumption, physical activity, diet, employment status, healthy sleep score, income levels, and education.
Abbreviations: Ref. reference group; *HR*, hazard ratio; FCRMD, first cardio-renal-metabolic disease; CRMM, cardio-renal-metabolic multimorbidity. Transition pattern A, transitions pathways based on four states in CRMM progression (see Figure 1B).

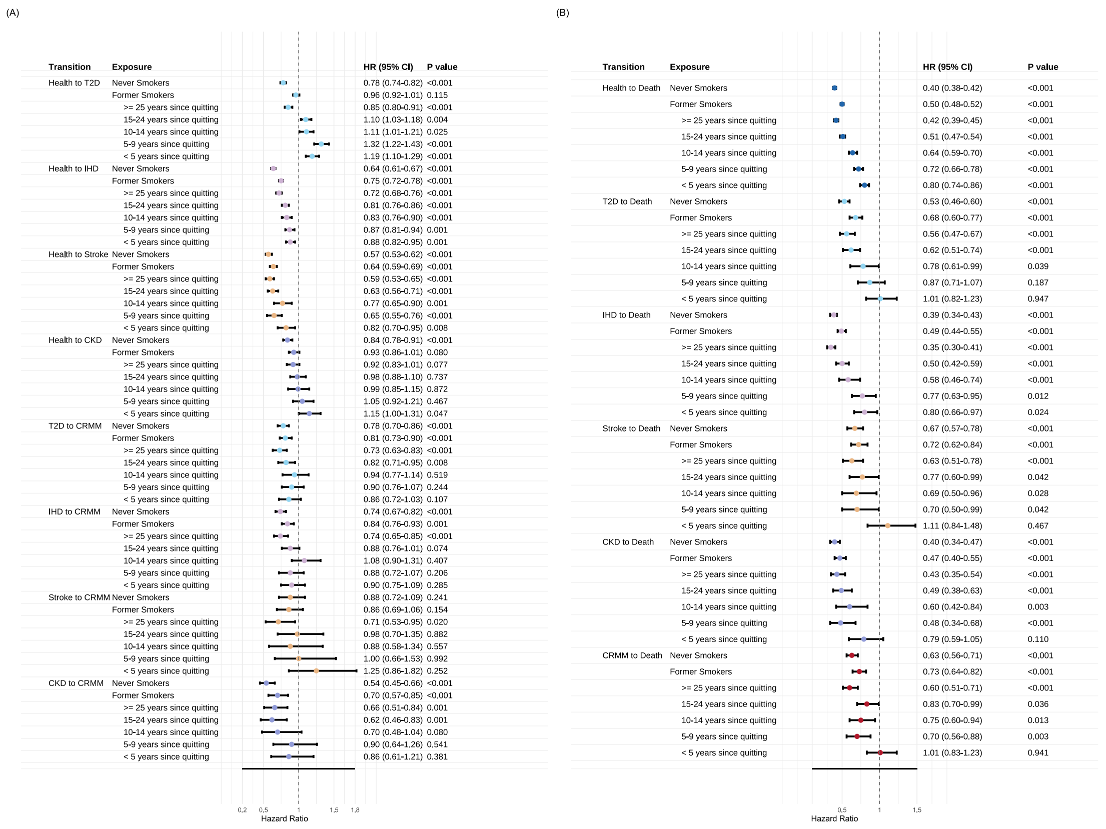

Figure S8 Association of smoking status and year since quitting with pathways in the cardio-renal-metabolic multimorbidity transition pattern B compared with current smokers in a prospective cohort study from the UK Biobank (baseline 2006–2010; follow-up through 2022).

Multi-state model was used to assess the role of smoking status in the temporal disease progression from baseline (free of CRMD) to each individual FCRMD, CRMM, and ultimately to death.

Abbreviations: *HR*, hazard ratio; *CI*, confidence interval; *P*, *P* value; Ref. reference group; IHD, ischemic heart disease; T2D, type 2 diabetes; CKD, chronic kidney disease; FCRMD, first cardio-renal-metabolic disease; CRMM, cardio-renal-metabolic multimorbidity. Transition pattern B, transitions pathways based on seven states in CRMM progression (see Figure 1C).

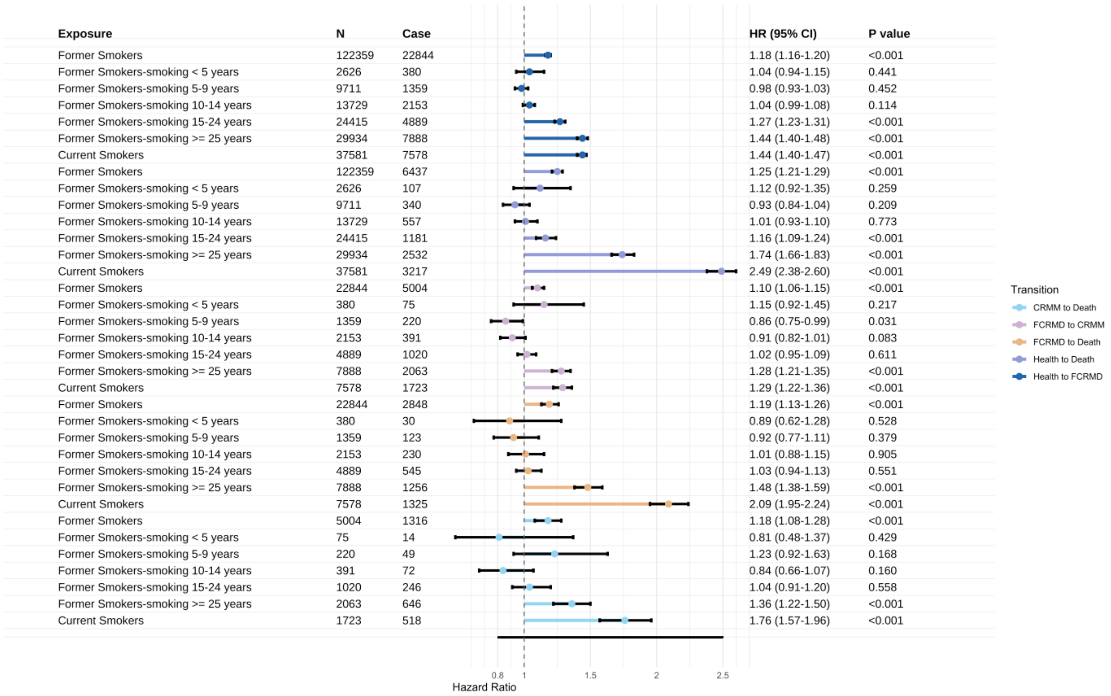

Figure S9 Association of smoking status and year of smoking before quitting with pathways in the cardio-renal-metabolic multimorbidity transition pattern comprising four states compared with never smokers in a prospective cohort study from the UK Biobank (baseline 2006–2010; follow-up through 2022).

Multi-state models adjusted for age, sex, alcohol consumption, physical activity, diet, employment status, healthy sleep score, income levels, and education.

Abbreviations: *N*, total sample size; Case, number of individuals experience the transition; *HR*, hazard ratio; *CI*, confidence interval; *P*, *P* value; FCRMD, first cardio-renal-metabolic disease; CRMM, cardio-renal-metabolic multimorbidity.

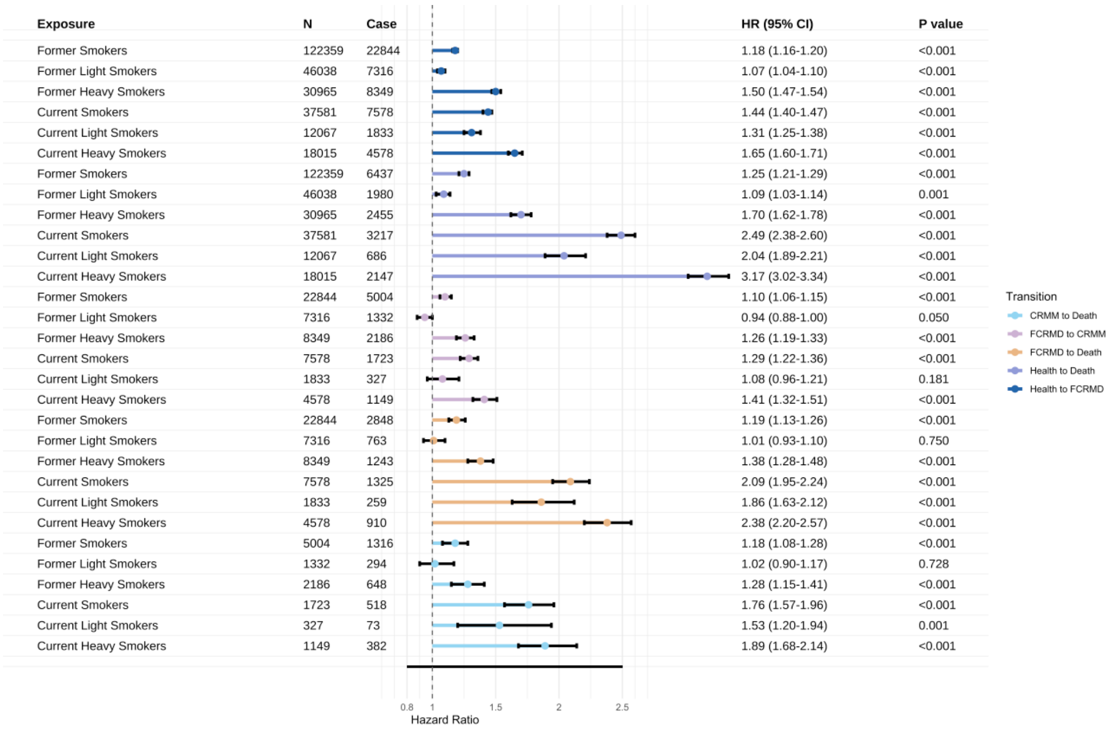

Figure S10 Association of smoking status and smoking intensity with pathways in the cardio-renal-metabolic multimorbidity transition pattern comprising four states compared with never smokers in a prospective cohort study from the UK Biobank (baseline 2006–2010; follow-up through 2022).

Multi-state models adjusted for age, sex, alcohol consumption, physical activity, diet, employment status, healthy sleep score, income levels, and education.

Abbreviations: *N*, total sample size; Case, number of individuals experience the transition; *HR*, hazard ratio; *CI*, confidence interval; *P*, *P* value; FCRMD, first cardio-renal-metabolic disease; CRMM, cardio-renal-metabolic multimorbidity.

Exposure	N	Case	HR (95% CI)	P value
Former Smokers	46038	7316	1.07 (1.04-1.10)	<0.001
Former Smokers- >= 25 years since quitting	24167	4624	1.03 (1.00-1.07)	0.039
Former Smokers-15-24 years since quitting	11485	1532	1.11 (1.05-1.17)	<0.001
Former Smokers-10-14 years since quitting	3921	451	1.15 (1.05-1.27)	0.003
Former Smokers-5-9 years since quitting	3416	394	1.20 (1.08-1.32)	<0.001
Former Smokers-< 5 years since quitting	3049	315	1.12 (1.01-1.26)	0.040
Current Smokers	12067	1833	1.31 (1.24-1.37)	<0.001
Former Smokers	46038	1980	1.09 (1.03-1.14)	0.001
Former Smokers- >= 25 years since quitting	24167	1240	1.01 (0.95-1.08)	0.699
Former Smokers-15-24 years since quitting	11485	398	1.12 (1.01-1.24)	0.030
Former Smokers-10-14 years since quitting	3921	147	1.52 (1.29-1.79)	<0.001
Former Smokers-5-9 years since quitting	3416	95	1.18 (0.96-1.44)	0.111
Former Smokers-< 5 years since quitting	3049	100	1.50 (1.23-1.83)	<0.001
Current Smokers	12067	686	2.04 (1.88-2.21)	<0.001
Former Smokers	7316	1332	0.94 (0.89-1.00)	0.062
Former Smokers- >= 25 years since quitting	4624	893	0.92 (0.86-0.99)	0.031
Former Smokers-15-24 years since quitting	1532	262	0.98 (0.86-1.11)	0.723
Former Smokers-10-14 years since quitting	451	72	0.99 (0.78-1.25)	0.918
Former Smokers-5-9 years since quitting	394	62	1.05 (0.82-1.35)	0.712
Former Smokers-< 5 years since quitting	315	43	0.94 (0.70-1.27)	0.702
Current Smokers	1833	327	1.06 (0.95-1.19)	0.290
Former Smokers	7316	763	1.01 (0.93-1.10)	0.737
Former Smokers- >= 25 years since quitting	4624	501	0.93 (0.85-1.03)	0.167
Former Smokers-15-24 years since quitting	1532	137	1.02 (0.86-1.21)	0.846
Former Smokers-10-14 years since quitting	451	55	1.60 (1.23-2.09)	0.001
Former Smokers-5-9 years since quitting	394	37	1.31 (0.94-1.81)	0.107
Former Smokers-< 5 years since quitting	315	33	1.63 (1.15-2.30)	0.006
Current Smokers	1833	259	1.86 (1.63-2.12)	<0.001
Former Smokers	1332	294	1.03 (0.90-1.17)	0.682
Former Smokers- >= 25 years since quitting	893	199	0.98 (0.84-1.14)	0.772
Former Smokers-15-24 years since quitting	262	61	1.26 (0.98-1.64)	0.076
Former Smokers-10-14 years since quitting	72	12	0.72 (0.41-1.27)	0.260
Former Smokers-5-9 years since quitting	62	11	1.02 (0.56-1.85)	0.944
Former Smokers-< 5 years since quitting	43	11	1.52 (0.84-2.76)	0.169
Current Smokers	327	73	1.48 (1.16-1.88)	0.002

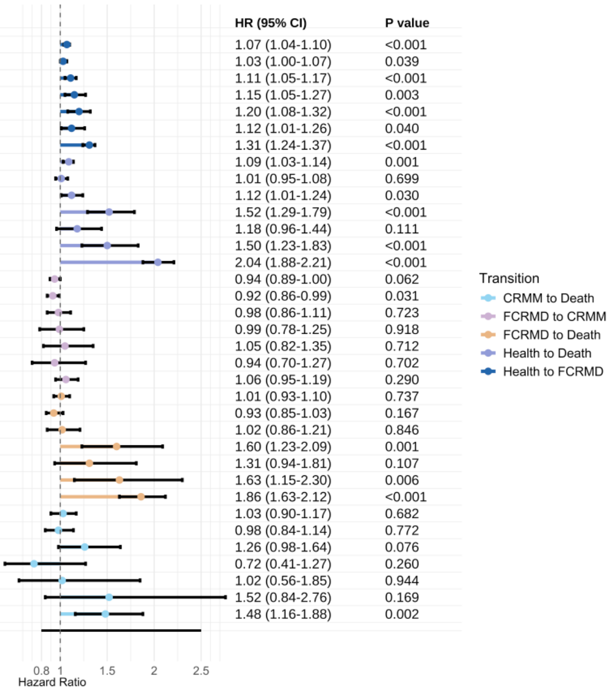

Figure S11 Association of smoking status and year since quitting with pathways in the cardio-renal-metabolic multimorbidity transition pattern comprising four states in light smokers (< 20 pack-year) compared with never smokers in a prospective cohort study from the UK Biobank (baseline 2006–2010; follow-up through 2022).

Multi-state models adjusted for age, sex, alcohol consumption, physical activity, diet, employment status, healthy sleep score, income levels, and education.

Abbreviations: *N*, total sample size; Case, number of individuals experience the transition; *HR*, hazard ratio; *CI*, confidence interval; *P*, *P* value; FCRMD, first cardio-renal-metabolic disease; CRMM, cardio-renal-metabolic multimorbidity.

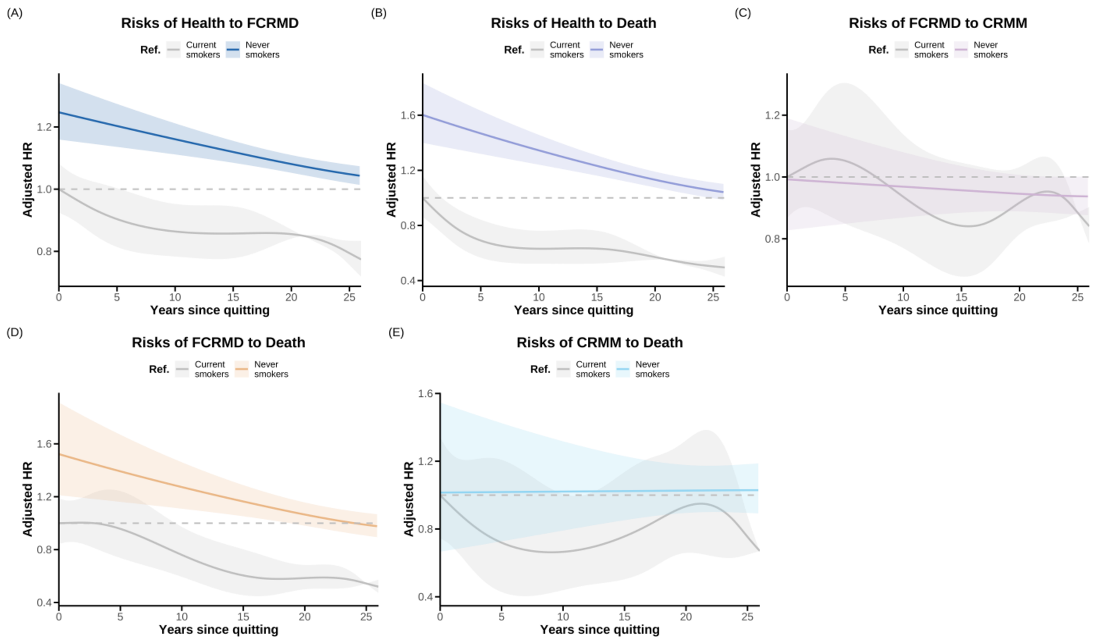

Figure S12 Risk for transition of pathways in the cardio-renal-metabolic multimorbidity transition pattern comprising four states by years since quitting in light smokers (< 20 pack-year) in a prospective cohort study from the UK Biobank (baseline 2006–2010; follow-up through 2022).

Restricted cubic splines with five knots were employed to capture potential nonlinear associations with the log hazard of the risk for transition of each pathway. Models were adjusted for age, sex, alcohol consumption, physical activity, diet, employment status, healthy sleep score, income levels, and education.

Abbreviations: *HR*, hazard ratio; Ref. reference group; FCRMD, first cardio-renal-metabolic disease; CRMM, cardio-renal-metabolic multimorbidity.

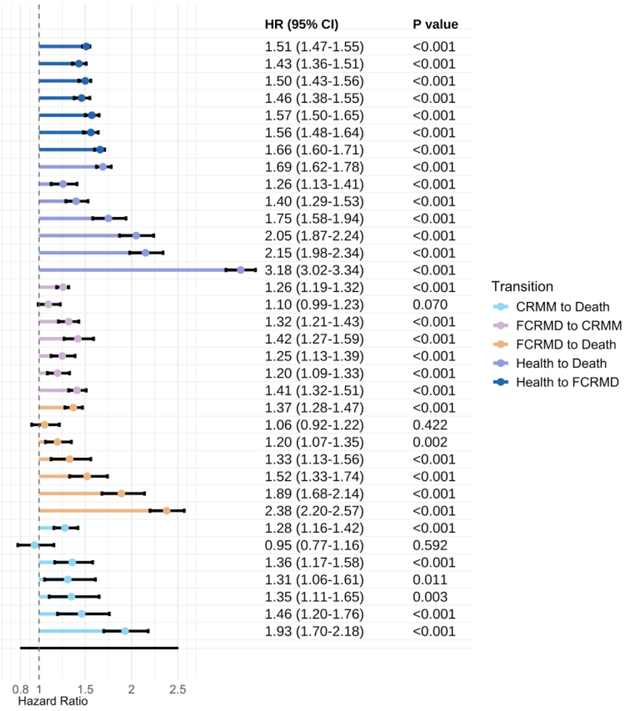

Exposure	N	Case	HR (95% CI)	P value
Former Smokers	30965	8349	1.51 (1.47-1.55)	<0.001
Former Smokers- >= 25 years since quitting	4332	1444	1.43 (1.36-1.51)	<0.001
Former Smokers-15-24 years since quitting	7979	2352	1.50 (1.43-1.56)	<0.001
Former Smokers-10-14 years since quitting	4711	1195	1.46 (1.38-1.55)	<0.001
Former Smokers-5-9 years since quitting	6499	1611	1.57 (1.50-1.65)	<0.001
Former Smokers-< 5 years since quitting	7444	1747	1.56 (1.48-1.64)	<0.001
Current Smokers	18015	4578	1.66 (1.60-1.71)	<0.001
Former Smokers	30965	2455	1.69 (1.62-1.78)	<0.001
Former Smokers- >= 25 years since quitting	4332	351	1.26 (1.13-1.41)	<0.001
Former Smokers-15-24 years since quitting	7979	592	1.40 (1.29-1.53)	<0.001
Former Smokers-10-14 years since quitting	4711	373	1.75 (1.58-1.94)	<0.001
Former Smokers-5-9 years since quitting	6499	539	2.05 (1.87-2.24)	<0.001
Former Smokers-< 5 years since quitting	7444	600	2.15 (1.98-2.34)	<0.001
Current Smokers	18015	2147	3.18 (3.02-3.34)	<0.001
Former Smokers	8349	2186	1.26 (1.19-1.32)	<0.001
Former Smokers- >= 25 years since quitting	1444	386	1.10 (0.99-1.23)	0.070
Former Smokers-15-24 years since quitting	2352	673	1.32 (1.21-1.43)	<0.001
Former Smokers-10-14 years since quitting	1195	333	1.42 (1.27-1.59)	<0.001
Former Smokers-5-9 years since quitting	1611	396	1.25 (1.13-1.39)	<0.001
Former Smokers-< 5 years since quitting	1747	398	1.20 (1.09-1.33)	<0.001
Current Smokers	4578	1149	1.41 (1.32-1.51)	<0.001
Former Smokers	8349	1243	1.37 (1.28-1.47)	<0.001
Former Smokers- >= 25 years since quitting	1444	210	1.06 (0.92-1.22)	0.422
Former Smokers-15-24 years since quitting	2352	327	1.20 (1.07-1.35)	0.002
Former Smokers-10-14 years since quitting	1195	162	1.33 (1.13-1.56)	<0.001
Former Smokers-5-9 years since quitting	1611	241	1.52 (1.33-1.74)	<0.001
Former Smokers-< 5 years since quitting	1747	303	1.89 (1.68-2.14)	<0.001
Current Smokers	4578	910	2.38 (2.20-2.57)	<0.001
Former Smokers	2186	648	1.28 (1.16-1.42)	<0.001
Former Smokers- >= 25 years since quitting	386	104	0.95 (0.77-1.16)	0.592
Former Smokers-15-24 years since quitting	673	213	1.36 (1.17-1.58)	<0.001
Former Smokers-10-14 years since quitting	333	99	1.31 (1.06-1.61)	0.011
Former Smokers-5-9 years since quitting	396	110	1.35 (1.11-1.65)	0.003
Former Smokers-< 5 years since quitting	398	122	1.46 (1.20-1.76)	<0.001
Current Smokers	1149	382	1.93 (1.70-2.18)	<0.001

Figure S13 Association of smoking status and year since quitting with pathways in the cardio-renal-metabolic multimorbidity transition pattern comprising four states in heavy smokers (>= 20 pack-year) compared with never smokers in a prospective cohort study from the UK Biobank (baseline 2006–2010; follow-up through 2022).

Multi-state models adjusted for age, sex, alcohol consumption, physical activity, diet, employment status, healthy sleep score, income levels, and education.

Abbreviations: *N*, total sample size; Case, number of individuals experience the transition; *HR*, hazard ratio; *CI*, confidence interval; *P*, *P* value; FCRMD, first cardio-renal-metabolic disease; CRMM, cardio-renal-metabolic multimorbidity.

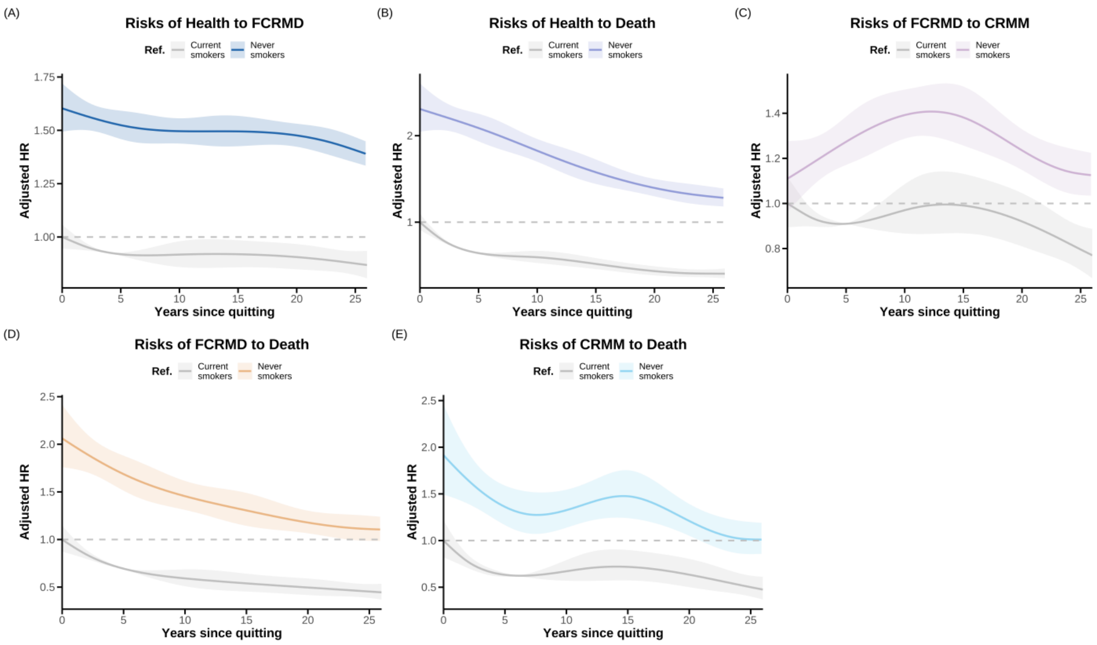

Figure S14 Risk for transition of pathways in the cardio-renal-metabolic multimorbidity transition pattern comprising four states by years since quitting in heavy smokers (>= 20 pack-year) in a prospective cohort study from the UK Biobank (baseline 2006–2010; follow-up through 2022).

Restricted cubic splines with five knots were employed to capture potential nonlinear associations with the log hazard of the risk for transition of each pathway. Models were adjusted for age, sex, alcohol consumption, physical activity, diet, employment status, healthy sleep score, income levels, and education.

Abbreviations: *HR*, hazard ratio; Ref. reference group; FCRMD, first cardio-renal-metabolic disease; CRMM, cardio-renal-metabolic multimorbidity.

Forest plot for low genetic risk and disease transitions

Transition	Smoking status	HR (95% CI)	P value
Health to T2D	Former Smokers	1.32 (1.19–1.47)	<0.001
Health to T2D	Former Smokers– >= 25 years since quitting	1.30 (1.11–1.54)	0.002
Health to T2D	Former Smokers–15–24 years since quitting	1.68 (1.40–2.01)	<0.001
Health to T2D	Former Smokers–10–14 years since quitting	1.28 (0.96–1.71)	0.094
Health to T2D	Former Smokers–5–9 years since quitting	1.85 (1.47–2.33)	<0.001
Health to T2D	Former Smokers–< 5 years since quitting	1.35 (1.04–1.74)	0.023
Health to T2D	Current Smokers	1.34 (1.14–1.56)	<0.001
Health to IHD	Former Smokers	1.21 (1.13–1.31)	<0.001
Health to IHD	Former Smokers– >= 25 years since quitting	1.15 (1.02–1.29)	0.021
Health to IHD	Former Smokers–15–24 years since quitting	1.27 (1.11–1.46)	0.001
Health to IHD	Former Smokers–10–14 years since quitting	1.31 (1.06–1.60)	0.011
Health to IHD	Former Smokers–5–9 years since quitting	1.52 (1.26–1.82)	<0.001
Health to IHD	Former Smokers–< 5 years since quitting	1.54 (1.29–1.84)	<0.001
Health to IHD	Current Smokers	1.59 (1.42–1.77)	<0.001
Health to Stroke	Former Smokers	1.26 (1.10–1.43)	0.001
Health to Stroke	Former Smokers– >= 25 years since quitting	1.17 (0.96–1.42)	0.130
Health to Stroke	Former Smokers–15–24 years since quitting	1.30 (1.02–1.65)	0.036
Health to Stroke	Former Smokers–10–14 years since quitting	1.15 (0.78–1.70)	0.474
Health to Stroke	Former Smokers–5–9 years since quitting	1.41 (1.01–1.97)	0.043
Health to Stroke	Former Smokers–< 5 years since quitting	1.85 (1.38–2.47)	<0.001
Health to Stroke	Current Smokers	2.08 (1.75–2.47)	<0.001
Health to CKD	Former Smokers	1.08 (0.97–1.19)	0.162
Health to CKD	Former Smokers– >= 25 years since quitting	1.01 (0.86–1.18)	0.915
Health to CKD	Former Smokers–15–24 years since quitting	1.28 (1.06–1.55)	0.010
Health to CKD	Former Smokers–10–14 years since quitting	0.94 (0.68–1.30)	0.696
Health to CKD	Former Smokers–5–9 years since quitting	1.31 (1.01–1.71)	0.042
Health to CKD	Former Smokers–< 5 years since quitting	1.43 (1.11–1.84)	0.006
Health to CKD	Current Smokers	1.15 (0.97–1.36)	0.100

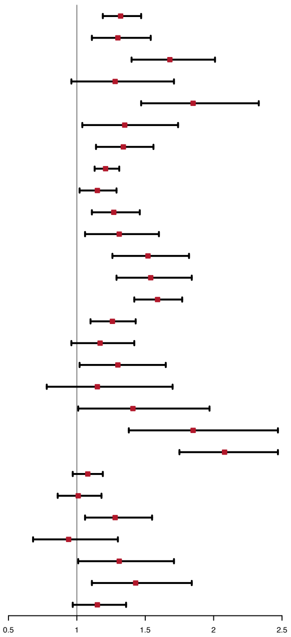

Forest plot for intermediate genetic risk and disease transitions

Transition	Smoking status	HR (95% CI)	P value
Health to T2D	Former Smokers	1.23 (1.18–1.29)	<0.001
Health to T2D	Former Smokers– >= 25 years since quitting	1.06 (0.99–1.14)	0.116
Health to T2D	Former Smokers–15–24 years since quitting	1.46 (1.35–1.57)	<0.001
Health to T2D	Former Smokers–10–14 years since quitting	1.49 (1.34–1.67)	<0.001
Health to T2D	Former Smokers–5–9 years since quitting	1.76 (1.60–1.94)	<0.001
Health to T2D	Former Smokers–< 5 years since quitting	1.57 (1.42–1.73)	<0.001
Health to T2D	Current Smokers	1.28 (1.20–1.36)	<0.001
Health to IHD	Former Smokers	1.17 (1.13–1.22)	<0.001
Health to IHD	Former Smokers– >= 25 years since quitting	1.14 (1.08–1.21)	<0.001
Health to IHD	Former Smokers–15–24 years since quitting	1.27 (1.19–1.36)	<0.001
Health to IHD	Former Smokers–10–14 years since quitting	1.33 (1.20–1.47)	<0.001
Health to IHD	Former Smokers–5–9 years since quitting	1.36 (1.24–1.50)	<0.001
Health to IHD	Former Smokers–< 5 years since quitting	1.39 (1.26–1.53)	<0.001
Health to IHD	Current Smokers	1.62 (1.54–1.71)	<0.001
Health to Stroke	Former Smokers	1.09 (1.01–1.18)	0.019
Health to Stroke	Former Smokers– >= 25 years since quitting	1.00 (0.89–1.12)	0.988
Health to Stroke	Former Smokers–15–24 years since quitting	1.12 (0.98–1.30)	0.106
Health to Stroke	Former Smokers–10–14 years since quitting	1.36 (1.12–1.66)	0.002
Health to Stroke	Former Smokers–5–9 years since quitting	1.14 (0.93–1.39)	0.210
Health to Stroke	Former Smokers–< 5 years since quitting	1.36 (1.13–1.64)	0.001
Health to Stroke	Current Smokers	1.67 (1.50–1.85)	<0.001
Health to CKD	Former Smokers	1.10 (1.03–1.17)	0.003
Health to CKD	Former Smokers– >= 25 years since quitting	1.14 (1.04–1.25)	0.005
Health to CKD	Former Smokers–15–24 years since quitting	1.13 (1.00–1.27)	0.042
Health to CKD	Former Smokers–10–14 years since quitting	1.28 (1.08–1.52)	0.004
Health to CKD	Former Smokers–5–9 years since quitting	1.13 (0.95–1.34)	0.166
Health to CKD	Former Smokers–< 5 years since quitting	1.35 (1.15–1.58)	<0.001
Health to CKD	Current Smokers	1.20 (1.08–1.32)	<0.001

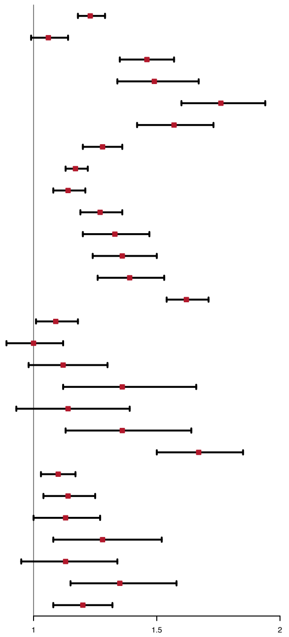

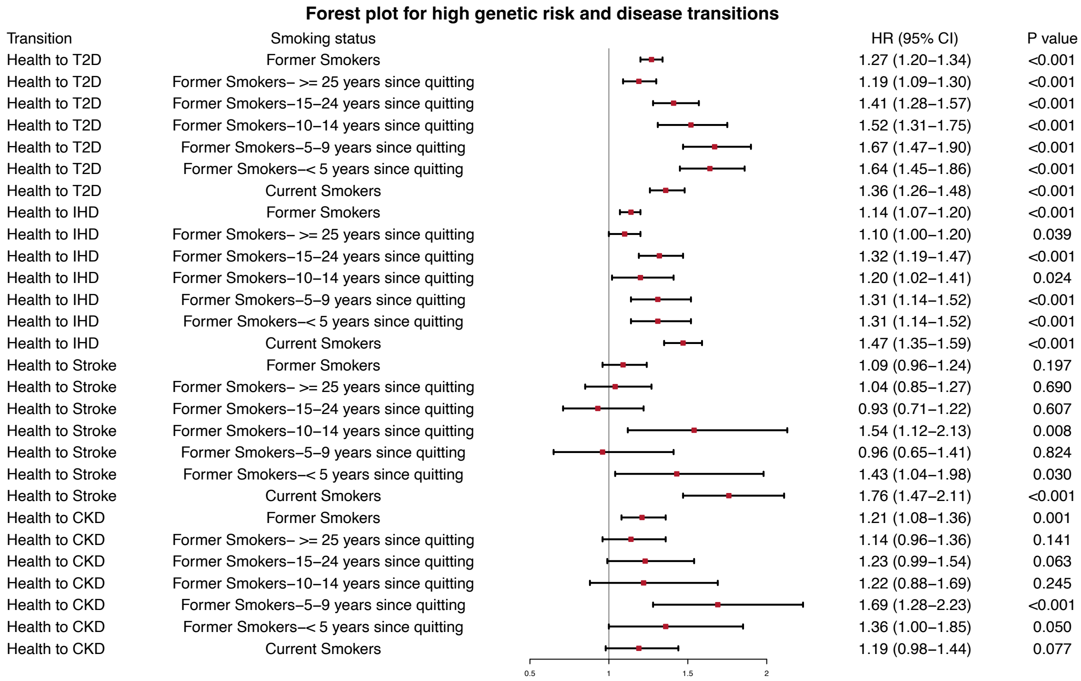

Figure S15 Association of smoking status and year since quitting with pathways from baseline to each individual first cardio-renal-metabolic disease in the cardio-renal-metabolic multimorbidity transition pattern comprising four states in subgroup with different genetic risk based on corresponding genetic risk score compared with never smokers in a prospective cohort study from the UK Biobank (baseline 2006–2010; follow-up through 2022).

Multi-state models adjusted for age, sex, alcohol consumption, physical activity, diet, employment status, healthy sleep score, income levels, and education.

All the participants were divided into high (quintile 5), intermediate (quintile 2–4), or low (quintile 1) genetic risk group based on weighted GRS for each individual disease.

Abbreviations: *N*, total sample size; Case, number of individuals experience the transition; *HR*, hazard ratio; *CI*, confidence interval; *P*, *P* value; IHD, ischemic heart disease; T2D, type 2 diabetes; FCRMD, first cardio-renal-metabolic disease; CRMM, cardio-renal-metabolic multimorbidity; GRS, genetic risk score.

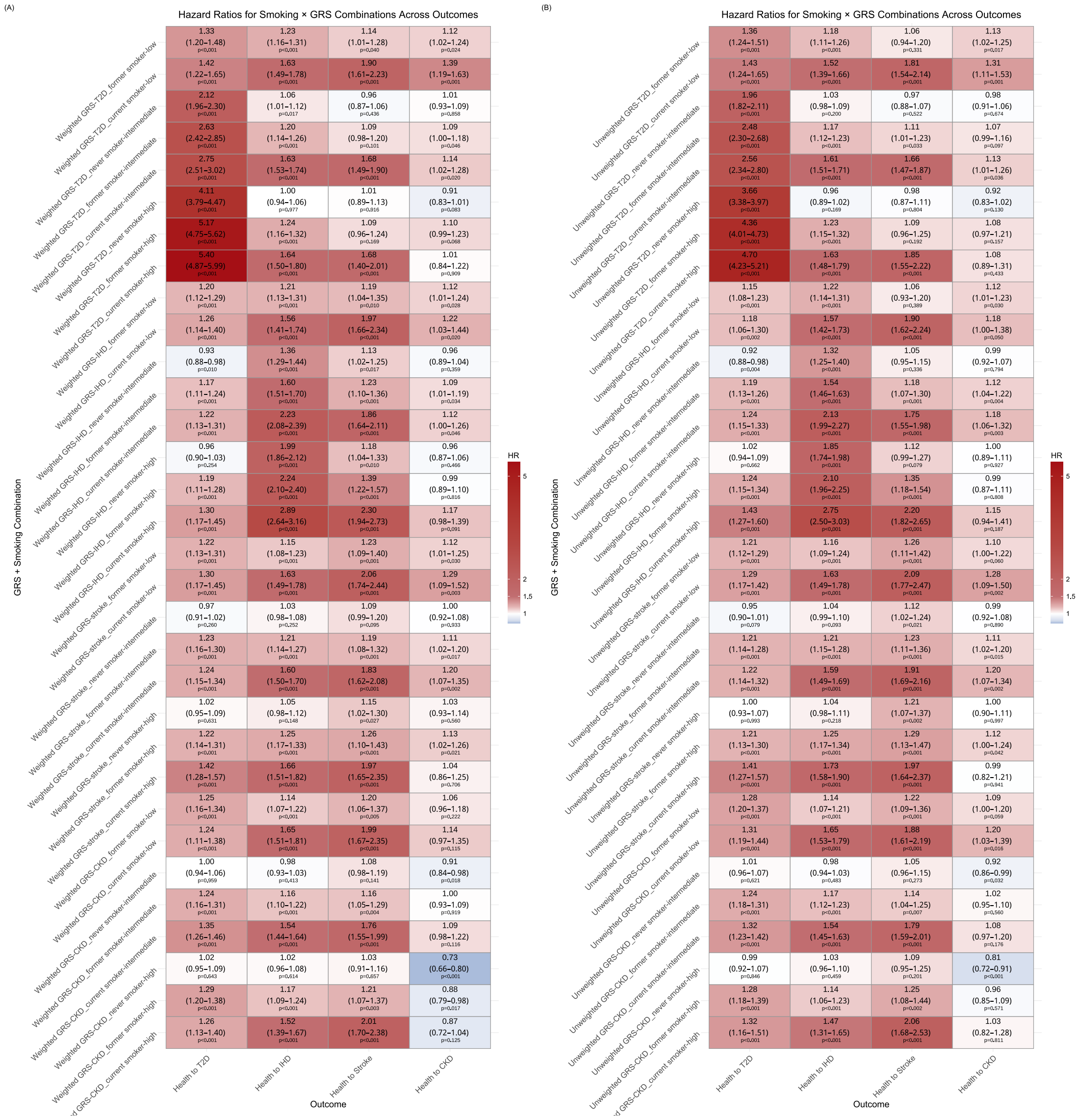

Figure S16 Joint effects of smoking status and year since quitting on pathways from baseline to each individual first cardio-renal-metabolic disease in the cardio-renal-metabolic multimorbidity transition pattern comprising four states compared with never smoker with low genetic risk in a prospective cohort study from the UK Biobank (baseline 2006–2010; follow-up through 2022).

Multi-state models adjusted for age, sex, alcohol consumption, physical activity, diet, employment status, healthy sleep score, income levels, and education.

All the participants were divided into high (quintile 5), intermediate (quintile 2–4), or low (quintile 1) genetic risk group based on weighted (A) or unweight (B) GRS for each individual disease.

Abbreviations: *N*, total sample size; Case, number of individuals experience the transition; *HR*, hazard ratio; *CI*, confidence interval; *p*, *P* value; IHD, ischemic heart disease; T2D, type 2 diabetes; FCRMD, first cardio-renal-metabolic disease; CRMM, cardio-renal-metabolic multimorbidity; GRS, genetic risk score.

References

1. Chudasama YV, Khunti KK, Zaccardi F, et al. Physical activity, multimorbidity, and life expectancy: a UK Biobank longitudinal study. *BMC Med*. 2019;17(1):108. doi:10.1186/s12916-019-1339-0
2. Ainsworth BE, Haskell WL, Whitt MC, et al. Compendium of physical activities: an update of activity codes and MET intensities. *Med Sci Sports Exerc*. 2000;32(9 Suppl):S498-504. doi:10.1097/00005768-200009001-00009
3. Carter AR, Harrison S, Gill D, et al. Educational attainment as a modifier for the effect of polygenic scores for cardiovascular risk factors: cross-sectional and prospective analysis of UK Biobank. *Int J Epidemiol*. 2022;51(3):885-897. doi:10.1093/ije/dyac002
4. Fan M, Sun D, Zhou T, et al. Sleep patterns, genetic susceptibility, and incident cardiovascular disease: a prospective study of 385 292 UK biobank participants. *Eur Heart J*. 2020;41(11):1182-1189. doi:10.1093/eurheartj/ehz849
5. Duncan MS, Freiberg MS, Greevy RA, Kundu S, Vasan RS, Tindle HA. Association of Smoking Cessation With Subsequent Risk of Cardiovascular Disease. *JAMA*. 2019;322(7):642-650. doi:10.1001/jama.2019.10298
6. Cho JH, Shin SY, Kim H, et al. Smoking Cessation and Incident Cardiovascular Disease. *JAMA Netw Open*. 2024;7(11):e2442639. doi:10.1001/jamanetworkopen.2024.42639
7. Nelson CP, Goel A, Butterworth AS, et al. Association analyses based on false discovery rate implicate new loci for coronary artery disease. *Nat Genet*. 2017;49(9):1385-1391. doi:10.1038/ng.3913
8. NINDS Stroke Genetics Network (SiGN), International Stroke Genetics Consortium (ISGC). Loci associated with ischaemic stroke and its subtypes (SiGN): a genome-wide association study. *Lancet Neurol*. 2016;15(2):174-184. doi:10.1016/S1474-4422(15)00338-5
9. Mahajan A, Spracklen CN, Zhang W, et al. Multi-ancestry genetic study of type 2 diabetes highlights the power of diverse populations for discovery and translation. *Nat Genet*. 2022;54(5):560-572. doi:10.1038/s41588-022-01058-3
10. Su J, Hu J, Liu H, et al. Causal role of the plasma lipidome in the occurrence and progression of chronic kidney disease: a two-sample Mendelian randomization study. *Diabetol Metab Syndr*. 2025;17(1):205. doi:10.1186/s13098-025-01764-y
11. Suzuki K, Hatzikotoulas K, Southam L, et al. Genetic drivers of heterogeneity in type 2 diabetes pathophysiology. *Nature*. 2024;627(8003):347-357. doi:10.1038/s41586-024-07019-6
12. Choi SW, Mak TSH, O'Reilly PF. Tutorial: a guide to performing polygenic risk score analyses. *Nat Protoc*. 2020;15(9):2759-2772. doi:10.1038/s41596-020-0353-1
13. Kurki MI, Karjalainen J, Palta P, et al. FinnGen provides genetic insights from a well-phenotyped isolated population. *Nature*. 2023;613(7944):508-518. doi:10.1038/s41586-022-05473-8
